# Cell cycle-independent furrowing triggered by phosphomimetic mutations of the INCENP STD motif requires Plk1

**DOI:** 10.1242/jcs.234401

**Published:** 2019-11-06

**Authors:** Diana Papini, Xavier Fant, Hiromi Ogawa, Nathalie Desban, Kumiko Samejima, Omid Feizbakhsh, Bilge Askin, Tony Ly, William C. Earnshaw, Sandrine Ruchaud

**Affiliations:** 1 Wellcome Centre for Cell Biology, University of Edinburgh, Max Born Crescent, Edinburgh EH9 3BF, Scotland, UK; 2 Sorbonne Université/CNRS UMR8227, Station Biologique, Place Georges Teissier, CS90074, 29688 ROSCOFF cedex, France

**Keywords:** CPC, INCENP, Furrow initiation, Aurora B, Plk1, Mitosis

## Abstract

Timely and precise control of Aurora B kinase, the chromosomal passenger complex (CPC) catalytic subunit, is essential for accurate chromosome segregation and cytokinesis. Post-translational modifications of CPC subunits are directly involved in controlling Aurora B activity. Here, we identified a highly conserved acidic STD-rich motif of INCENP that is phosphorylated during mitosis *in vivo* and by Plk1 *in vitro* and is involved in controlling Aurora B activity. By using an INCENP conditional-knockout cell line, we show that impairing the phosphorylation status of this region disrupts chromosome congression and induces cytokinesis failure. In contrast, mimicking constitutive phosphorylation not only rescues cytokinesis but also induces ectopic furrows and contractile ring formation in a Plk1- and ROCK1-dependent manner independent of cell cycle and microtubule status. Our experiments identify the phospho-regulation of the INCENP STD motif as a novel mechanism that is key for chromosome alignment and cytokinesis.

This article has an associated First Person interview with the first author of the paper.

## Abstract (precis)

**Highlighted Article:** Phospho-regulation of the INCENP STD motif acts as a novel switch that is key to Aurora B activity modulation, chromosome alignment and cytokinesis.

## INTRODUCTION

Mitosis and cytokinesis are coordinated by essential kinases whose expression and activity are deregulated in many cancers (Li and Li, 2006; Malumbres and Barbacid, 2007). Cyclin-dependent kinase-1 (CDK1), Aurora A and Polo-like kinase 1 (Plk1) cooperate to regulate mitotic entry (Lindqvist et al., 2009). Subsequently, Plk1 and Aurora B direct bipolar spindle formation, spindle stability, chromosome congression and bi-orientation (Sunkel and Glover, 1988; Sampath et al., 2004; Sumara et al., 2004; Lénárt et al., 2007; Petronczki et al., 2008). At anaphase onset, as CDK1 activity drops, both Aurora B and Plk1 control cytokinesis (Ruchaud et al., 2007; Petronczki et al., 2008; Carmena et al., 2009; Hümmer and Mayer, 2009; Carmena et al., 2012; van der Horst and Lens, 2014).

Cytokinesis is the process by which cells divide to yield two daughter cells (Pollard and O'Shaughnessy, 2019). Cytokinesis initiates with cleavage furrow ingression mediated by the action of an acto-myosin contractile ring triggered by local membrane activation of the small GTPase RhoA (D'Avino et al., 2005; Piekny et al., 2005; D'Avino and Glover, 2009). RhoA drives contractile ring assembly through the activation of formin actin assembly factors and ROCK1 kinase, which subsequently phosphorylates members of the myosin regulatory light chain (MRLC) family (Matsumura, 2005; Otomo et al., 2005). RhoA activation requires recruitment of the active form of the RhoGEF Ect2 to the plasma membrane (Wagner and Glotzer, 2016; Basant and Glotzer, 2018) by the centralspindlin complex composed of the kinesin-like protein MKLP1 (also known as KIF23) and the RhoGAP Cyk4 (also known as MgcRacGAP and RACGAP1) (Mishima et al., 2002; Yüce et al., 2005; Zhao and Fang, 2005; Nishimura and Yonemura, 2006; Pavicic-Kaltenbrunner et al., 2007; Lekomtsev et al., 2012).

Plk1-mediated phosphorylation of Cyk4 is essential for cleavage furrow ingression and cytokinesis in part by creating a binding site for the BRCT domain of Ect2 (Burkard et al., 2007, 2009; Petronczki et al., 2007; Wolfe et al., 2009). It also prevents premature midzone formation by regulating the activity of the microtubule-bundling protein PRC1 (Hu et al., 2012). Ultimately, Plk1 phosphorylation of PRC1 releases centralspindlin from the central spindle allowing its recruitment at the plasma membrane (Adriaans et al., 2019).

Aurora B kinase regulates many aspects of mitosis ranging from chromosome and spindle structure to the correction of kinetochore–microtubule attachment errors, regulation of mitotic progression and completion of cytokinesis (Vagnarelli and Earnshaw, 2004; Vader et al., 2006; Ruchaud et al., 2007; Carmena et al., 2012; van der Horst and Lens, 2014; D'Avino et al., 2015). Aurora B is part of the chromosomal passenger complex (CPC) composed of INCENP (Cooke et al., 1987; Terada et al., 1998; Adams et al., 2000), survivin (also known as BIRC5) and Borealin (also known as Dasra B and CDCA8) (Honda et al., 2003; Gassmann et al., 2004; Sampath et al., 2004; Klein et al., 2006). INCENP is required for Aurora B activation via direct binding and a phosphorylation feedback loop (Bishop and Schumacher, 2002; Sessa et al., 2005; Ruchaud et al., 2007), and, with Survivin and Borealin, forms a localization module for the CPC (Jeyaprakash et al., 2007; Kelly et al., 2010; Wang et al., 2010; Yamagishi et al., 2010). Knockdown by RNA interference (RNAi) of any CPC member delocalizes the others and disrupts spindle midzone transfer and cytokinesis (Adams et al., 2001; Carvalho et al., 2003; Honda et al., 2003; Lens et al., 2003; Gassmann et al., 2004; Vader et al., 2006). Aurora B phosphorylation of the centralspindlin component MKLP1 releases it from inhibition by the PAR5 protein (14-3-3 family in mammals) and allows it to oligomerize with Cyk4 (Minoshima et al., 2003; Guse et al., 2005; Basant et al., 2015).

Even prior to discovery of the CPC, INCENP was suggested to have a role in the initiation of membrane furrowing, as it could be detected at the equatorial cortex in anaphase before any detectable furrowing and before myosin had begun to concentrate in a contractile ring (Earnshaw and Cooke, 1991; Eckley et al., 1997). However, it was later suggested that INCENP and the CPC may not be essential for furrow initiation during mitotic exit (Adams et al., 2001; Guse et al., 2005; Ahonen et al., 2009). All studies agree that CPC activity is required to complete cytokinesis (Schumacher et al., 1998; Tatsuka et al., 1998; Terada et al., 1998; Adams et al., 2001; Honda et al., 2003; Gassmann et al., 2004; Vader et al., 2006; Yue et al., 2008; Xu et al., 2009), where it plays a key role in regulating the final events of abscission (Capalbo et al., 2016; Pike et al., 2016).

To better understand the role of INCENP in regulating mitosis and cytokinesis, we have performed a functional analysis of conserved serine and threonine sites on the INCENP polypeptide that are specifically phosphorylated during mitosis. Here, we describe a highly conserved negatively charged region located in the N-term of INCENP IN-box whose phosphorylation is essential for cytokinesis and chromosome alignment. Surprisingly, we show that mimicking constitutive phosphorylation of this domain triggers constitutive Plk1-dependent cell cycle-independent contractile ring assembly and ectopic furrow formation.

## RESULTS

### Phosphorylation on INCENP during mitosis

**Fig. 1.**
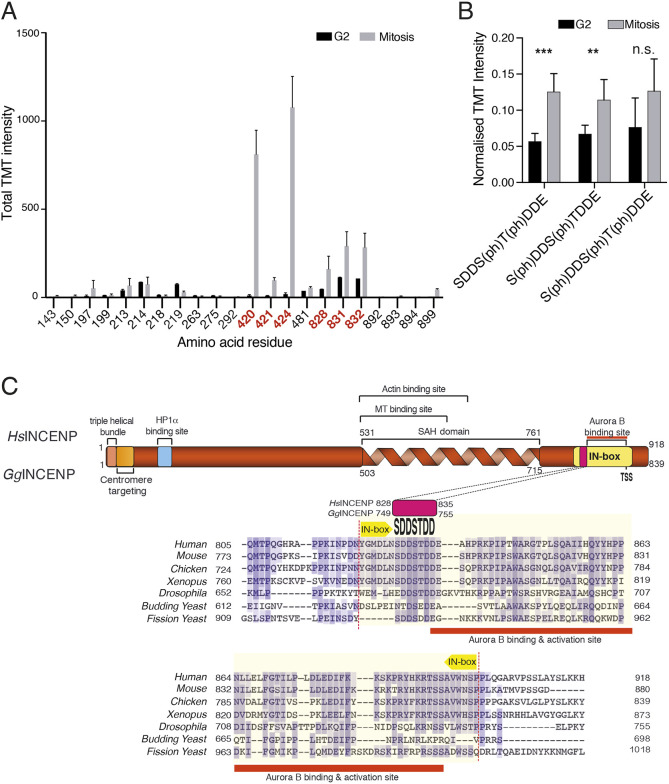
**INCENP is highly phosphorylated during mitosis at S420, T424, S828, S831, and T832.** (A) MS-based TMT quantification of INCENP phosphorylation sites comparing arrested G2 cells versus mitotic TK6 CDK1 using PRIMMUS (see Materials and Methods; Ly et al., 2017). TMT intensities are summed by phosphorylated residue. Residues that show phosphorylation changes with *P*<0.05 are highlighted in red [*n*=3, false discovery rate (FDR) corrected *t*-test]. (B) Data from phosphopeptides that span S828/S831/T832 residues of INCENP. *P*-values from FDR-corrected *t*-tests are shown above each peptide (*n*=3). (ph) indicates that the preceding residue is the phosphoacceptor site. (C) Schematic representation of INCENP protein highlighting specific domains of interest. The lower panel shows a ‘Muscle’ alignment of C-terminal parts of INCENP orthologs. The percentage of identity is color coded. The residues whose phosphorylation is upregulated during mitosis are set in a highly conserved region [pink box, amino acid numbering is shown for *Homo sapiens* (Hs) and *Gallus gallus* (*Gg*) INCENP] and localized upstream of the Aurora B-binding site (red line) within the IN-box (light yellow). n.s., not significant, *P*>0.05; **P≤0.01; ***P≤0.001.

Phosphoproteomics analyses have previously shown that a conserved motif near the N-terminal end of the INCENP IN-box is phosphorylated during mitosis while INCENP is on the spindle (Nousiainen et al., 2006; Dephoure et al., 2008; Malik et al., 2009; Oppermann et al., 2009). We used a recently described cell cycle fractionation procedure (Ly et al., 2017) to confirm that phosphorylation of this region is significantly increased in flow-sorted mitotic human TK6 cells that were histone H3 serine 28 phosphorylation (H3S28ph)-high, CycA+ and CycB+ (i.e. in early prometaphase; Fig. 1A,B; Fig. S1). Our analysis identified 22 phosphorylation sites on human (*Hs*)INCENP. Two sites at amino acids 420 and 424 show a particularly marked increase in phosphorylation. These two sites are not conserved in chicken INCENP, so we turned our attention to three other sites at S828, S831 and T832 (S749, S752, T753 in chicken INCENP class I) that also show significantly increased phosphorylation during mitosis. This very highly conserved region lies just within the IN-box, as originally defined (Adams et al., 2000), and is adjacent to the portion of the IN-box shown to bind Aurora B (Sessa et al., 2005) (Fig. 1C).

### Phosphorylation on INCENP residues S752 and T753 regulates Aurora B activity and chromosome alignment

**Fig. 2.**
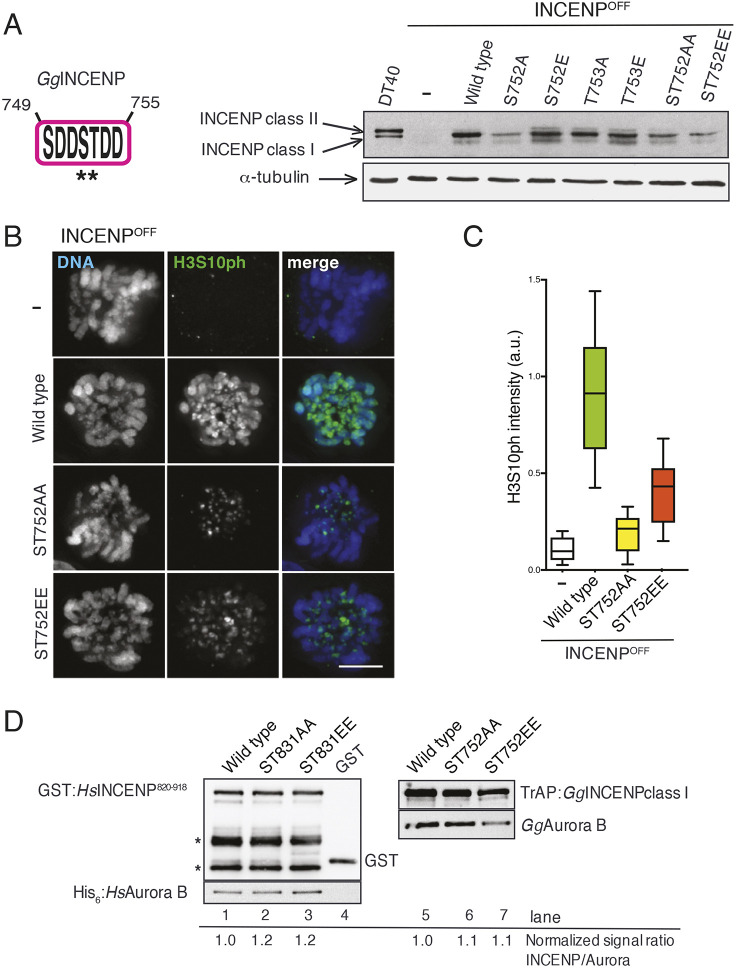
**Phosphomimetic and phosphodeficient mutations on S752 and/or T753 of INCENP affect H3S10 phosphorylation but do not affect Aurora B binding.** (A) Left, *Gg*INCENP STD motif diagram. Asterisks represent the two amino acids that were mutated to either alanine or glutamic acid. Right, immunoblot showing depletion of endogenous INCENP class I and II proteins in the INCENP-knockout cells (INCENP^OFF^) compared with endogenous protein expression in DT40 cells together with the expression levels of exogenous INCENP class I wild type or specific single or double mutations on S752 and T753 in the INCENP^OFF^ background, 24 h after addition of doxycycline. (B) Representative immunostaining of H3S10ph (green) and DNA (DAPI, blue) in INCENP^OFF^ and INCENP^OFF^ and INCENP^OFF^ cells expressing wild-type, ST752AA and ST752EE INCENP mutant proteins. Images were acquired using the same microscope settings. Scale bar: 5 µm. (C) Quantification of H3S10ph signal on prometaphase INCENP^OFF^ cells and INCENP^OFF^ cells expressing wild-type, ST752AA and ST752EE INCENP mutant proteins (*n*>12). The box represents the 25–75th percentiles, and the median is indicated. The whiskers show the minimum and maximum values. (D) Left panel (lanes 1–4), immunoblot of GST pulldowns on GST-tagged *Hs*INCENP C-terminal bearing or not double mutations ST831AA or ST831EE; *Hs*Aurora B levels are shown alongside normalized INCENP:Aurora B ratios. Bands corresponding to proteolytic INCENP fragments are indicated with asterisks. Right panel (lanes 5–7), streptavidin pulldowns on extracts from *Gg*INCENP^OFF^ cells expressing SBP-tagged wild-type, ST752AA or ST752EE *Gg*INCENP mutant proteins showing *Gg*Aurora B protein levels.

In order to determine the function of these phosphorylation events in mitotic regulation by the CPC, we expressed phosphodeficient and phosphomimetic forms of chicken (*Gg*)INCENP class I protein mutated at S749, S752 and T753 in an INCENP conditional (tet-off) knockout prepared in chicken DT40 (B-lymphoblastoid) cells (protocol in Fig. S2A) (Samejima et al., 2008; Xu et al., 2009). Here, we refer to cells with the genotype *INCENP^c/−^* growing normally in culture as INCENP^ON^. ‘c’ is a conditional allele of INCENP in which the promoter has been hijacked so that both INCENP class I and II isoforms are expressed from the endogenous gene, but under tetracycline control (Samejima et al., 2008; Xu et al., 2009). [Chicken INCENP has two isoforms that differ by the insertion of 38 residues near the C′ end of the SAH domain. Either isoform can support life in DT40 cells (Mackay et al., 1993)]. We refer to the cells as INCENP^OFF^ when they are grown in the presence of doxycycline for a minimum of 24 h, by which time INCENP protein becomes undetectable in immunoblots (Fig. 2A; Fig. S2A, lane 2). Stable *INCENP^c/−^* clones expressing the mutant proteins were isolated. We selected clones in which, after shutoff of the conditional endogenous allele, the mutant proteins were expressed at levels similar to INCENP in wild-type DT40 cells for subsequent analysis (Fig. 2A). Addition of doxycycline allowed us to analyze the behavior of each mutant in an INCENP-null background. Cells expressing the S749A mutant were normal in all assays tested, so this site is not discussed further here.

Immunofluorescence analysis of H3S10ph levels on prometaphase chromosomes from the various mutant cell lines suggested that Aurora B activity *in vivo* was reduced when INCENP was mutated on S752 and T753 (Fig. 2B,C; Fig. S2B). INCENP^OFF^ cells expressing INCENP^S752A,T753A^ (from here on abbreviated INCENP^ST752AA^) showed significantly reduced levels of H3S10ph similar to INCENP^OFF^ cells. In contrast, INCENP^OFF^ cells expressing the double phosphomimetic INCENP^S752E,T753E^ (from here on abbreviated INCENP^ST752EE^) exhibited ∼50% of H3S10ph levels of cells expressing exogenous INCENP^WT^ (Fig. 2B,C).

**Fig. 3.**
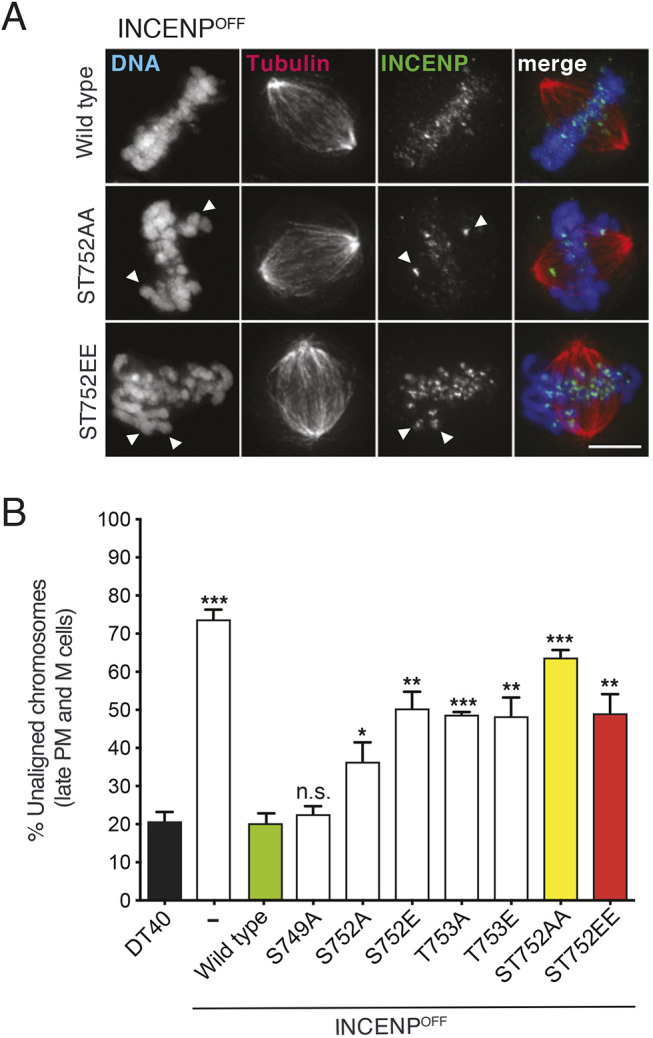
**Controlling phosphorylation on S752 and T753 is required for chromosome alignment*.*** (A) Immunostaining of INCENP (green) and α-tubulin (red) together with DNA (DAPI, blue) on INCENP^OFF^ metaphase cells expressing INCENP wild-type, ST752AA or ST752EE mutant proteins. Arrowheads highlight misaligned chromosomes. Scale bar: 5 µm. (B) Quantification of the proportion of late prometaphase (PM) and metaphase (M) cells showing unaligned chromosomes in DT40 cells, INCENP^OFF^ and INCENP^OFF^ cells expressing single and double phosphodeficient and phosphomimetic mutant INCENP proteins. Results are mean±s.e.m. (*n*=3 independent experiments). *P≤0.05; **P≤0.01; ***P≤0.001; n.s., not significant, *P*>0.05 (two-tailed unpaired *t*-test).

The decreased H3S10 phosphorylation did not result from a lack of Aurora B binding by the various INCENP mutants or from incorrect localization of the CPC. GST pulldowns with baculovirus-expressed human His-tagged Aurora B and bacterially expressed wild-type and mutant human GST–INCENP peptides showed that *in vitro*, similar levels of Aurora B were bound to a C-terminal peptide from INCENP wild type (aa 820–918), INCENP^ST831AA^ or INCENP^ST831EE^ (Fig. 2D, lanes 1–3). This was confirmed *in vivo* by using DT40 INCENP^OFF^ cells expressing triple affinity purification (TrAP)-tagged (Hudson et al., 2008) full-length chicken INCENP (wild type, the INCENP^ST752AA^ or INCENP^ST752EE^ mutants). Similar amounts of Aurora B kinase were pulled down in all cases (Fig. 2D, lanes 5–7). Thus, defects in Aurora B binding cannot explain the lower H3S10ph levels seen in cells expressing the INCENP mutations. Consistent with these observations, these INCENP mutants localized normally to centromeres during metaphase (Fig. 3A).

INCENP depletion severely disrupted chromosome alignment, with more than 70% of early mitotic cells having non-aligned chromosomes (Fig. 3A,B). All phosphodeficient and phosphomimetic single and double mutants failed to rescue chromosome alignment in the INCENP^OFF^ background, with the exception of INCENP^S749A^ and to a lesser extent the INCENP^S752A^ mutant (between 35 and 64% misalignment compared to 21% for rescue by exogenous INCENP^WT^; Fig. 3B). The phosphodeficient INCENP^ST752AA^ double mutant showed the most severe effects, closely resembling the INCENP^OFF^ cells (64% and 74% misalignment, respectively). Thus, controlled phosphorylation of these residues is required for chromosome alignment.

This region of INCENP is also implicated in normal function of the spindle assembly checkpoint (SAC) in DT40 cells. The involvement of the CPC in the SAC in DT40 cells appears to be less prominent than it is in cell lines from other vertebrates (Yue et al., 2008; Xu et al., 2009), nonetheless loss of INCENP causes a significant decrease in mitotic index in cells exposed to low doses of taxol (Fig. S2C). This checkpoint defect was also seen in INCENP^OFF^ cells expressing INCENP^ST752AA^ and INCENP^ST752EE^. An example of an INCENP^ST752EE^-expressing cell entering anaphase with an unaligned chromosome is shown in Fig. S2D.

Taken together, our results suggest that phosphorylation of INCENP on S752 and T753 represents a novel mechanism regulating Aurora B activity *in vivo* that is necessary for normal chromosome alignment and checkpoint function in early mitosis. We refer to this conserved domain of INCENP as the STD motif. This highly negatively charged motif at the N-terminal end of the IN-box is the most highly conserved region of the INCENP polypeptide.

### Phosphorylation of INCENP on both S752 and T753 is required for cytokinesis

**Fig. 4.**
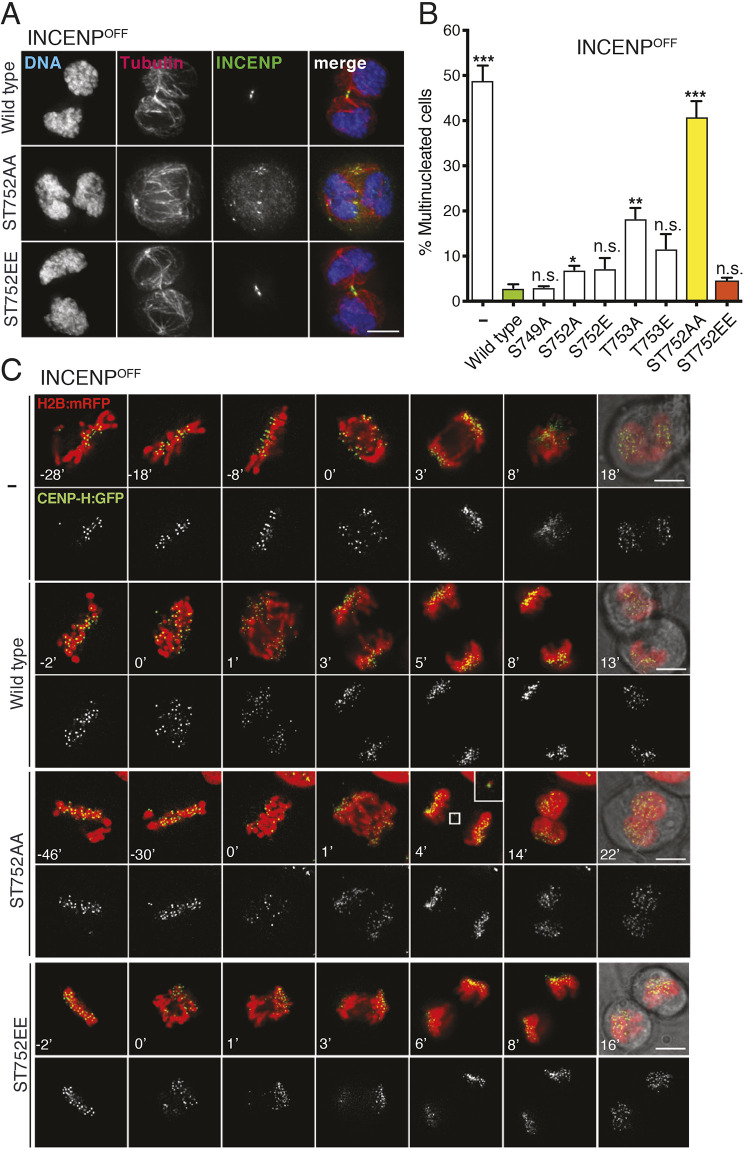
**Phosphorylation on S752 and T753 are synergistically required for cytokinesis.** (A) Immunostaining of INCENP (green) and α-tubulin (red) together with DNA (DAPI, blue) on INCENP^OFF^ late telophase cells expressing INCENP wild-type, ST752AA or ST752EE mutant proteins. Scale bar: 5 µm. (B) Quantification of the proportion of multinucleated INCENP^OFF^ and INCENP^OFF^ cells expressing various single and double phosphodeficient and phosphomimetic mutant INCENP proteins. Results are mean±s.e.m. (*n*=3 independent experiments). *P≤0.05; **P≤0.01; ***P≤0.001; n.s., not significant, *P*>0.05 (two-tailed unpaired *t*-test). (C) Chosen frames (time in minutes relative to anaphase onset, set at 0 min) from time-lapse live-cell imaging of INCENP^OFF^ cells and INCENP^OFF^ cells expressing wild-type, ST752AA or ST752EE INCENP mutant proteins stably expressing H2B–mRFP (H2B:mRFP, red) and CENP-H–GFP (CENP-H:GFP, green). CENP-H–GFP signal (lower frames) and merged images are shown. For each movie, the last frame is shown super-imposed with the phase-contrast image. Scale bars: 5 µm.

As we reported previously, INCENP^OFF^ cells suffer profound defects in cytokinesis. This can be observed through an increase of multinucleated cells in fixed samples and by time-lapse live-cell imaging (Xu et al., 2009) (Fig. 4A–C; Movies 1–4). By 26 h in doxycycline, 48% of surviving INCENP^OFF^ cells were bi- or multi-nucleated, compared with 3% for INCENP^OFF^ cells expressing INCENP^WT^ (Fig. 4B). INCENP^OFF^ cells expressing phosphodeficient INCENP^ST752AA^ showed failed cytokinesis to a degree similar to INCENP^OFF^ cells. Consistent with this, localization of INCENP^ST752AA^ appeared to be defective during mitotic exit – the protein was either diffuse throughout the cell or present in lower than normal amounts across the midzone (Fig. S3A). Nonetheless, these cells appeared to assemble spindles with midzones capable of supporting anaphase B spindle elongation, despite the reduced levels of INCENP (Fig. S4, Movies 3, 5 and 6). Comparison of multinucleation indexes seen with phosphodeficient single mutations INCENP^S752A^ (7%), INCENP^T753A^ (18.3%) and the double mutation INCENP^ST752AA^ (41%) suggested that phosphorylation on these two residues acts in a synergistic manner (Fig. 4B).

Remarkably, the INCENP^ST752EE^ double phosphomimetic mutant fully rescued cytokinesis in INCENP^OFF^ cells, with levels of multinucleation similar to wild type (4.6% and 3%, respectively; Fig. 4B). The localization of the mutant protein appeared to be normal late in mitotic exit, although we could occasionally observe an apparent delay in INCENP transfer from the chromosomes to the central spindle early in anaphase (Fig. S3A). As expected given the lower levels of Aurora B activity (H3S10ph) observed in INCENP^OFF^ cells expressing INCENP^ST752EE^, cytokinesis in these cells was more sensitive to Aurora B inhibition with ZM447439 than in wild type. Treatment with 100 nM ZM4437439 was sufficient to induce binucleation in INCENP^ST752EE^ cells whereas wild-type cells were largely unaffected by treatment with this inhibitor concentration (Fig. S3B,C).

Our data show that despite its correct localization to centromeres in early mitosis, INCENP^ST752EE^ does not enable the CPC to correct chromosome attachment errors but is sufficient to allow spindle transfer and cytokinesis. The low level of multi-nucleated cells in cultures of INCENP^OFF^ cells expressing INCENP^ST752EE^ also indicates that furrowing driven by this mutant can apparently proceed to completion in mitosis.

### INCENP^ST752EE^ triggers formation of cell cycle-independent ectopic furrows

**Fig. 5.**
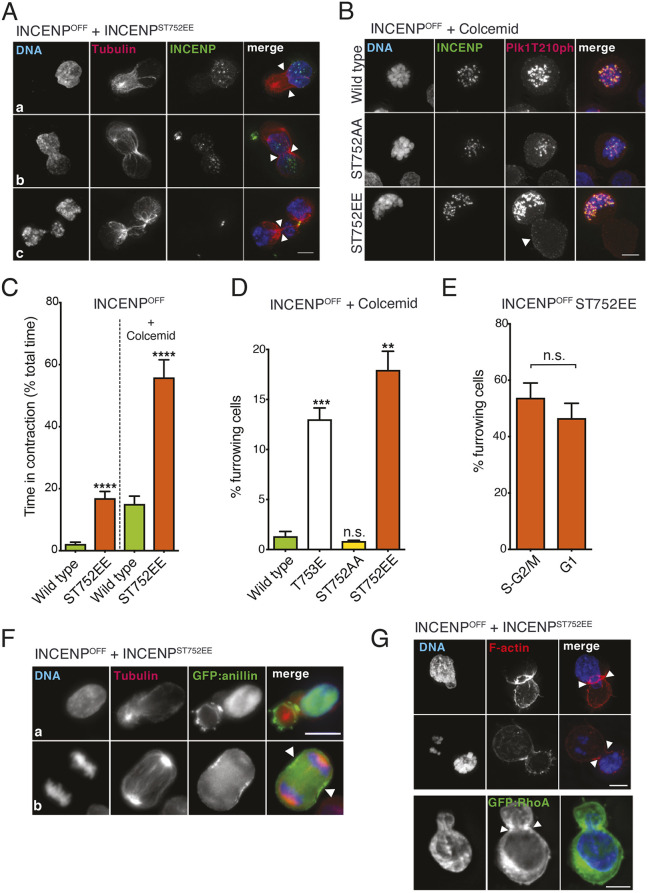
**ST752EE mutant triggers the formation of ectopic cleavage furrows.** (A) Immunostaining of INCENP (green) and α-tubulin (red) together with DNA (DAPI, blue) on INCENP^OFF^ cells expressing INCENP ST752EE mutant protein. White arrowheads are pointing at ectopic cleavage furrow in formation where INCENP is not recruited. Cells in interphase (a), prometaphase (b) and cytokinesis (c) are shown. (B) Immunostaining of INCENP (green) and Plk1T210ph (red) together with DNA (DAPI, blue) on colcemid-treated INCENP^OFF^ cells expressing wild-type, ST752AA and ST752EE INCENP mutant proteins. White arrowheads are pointing at ectopic membrane contractions. (C) Measurement of time in contraction from DIC movies (as a percentage of total movie time) on INCENP^OFF^ cells expressing INCENP wild-type or ST752EE mutation in the presence or absence of colcemid (left graph); a minimum of 18 cells per condition were analyzed. (D) Quantification of furrowing events in INCENP^OFF^ cells, treated in colcemid expressing wild-type, ST752AA, T753E and ST752EE mutant proteins. (E) Quantification of furrowing events in INCENP^OFF^ cells expressing INCENP ST752EE in S-G2 and G1 phase of the cell cycle. Results in C–E are mean±s.e.m. [*n*=3 (C,D) or *n*=2 independent experiments (E)]. **P≤0.01; ***P≤0.001; n.s., not significant, *P*>0.05 (two-tailed unpaired *t*-test). (F) Immunostaining of α-tubulin (red) together with DNA (DAPI, blue) in GFP–anillin (GFP:anillin)-expressing INCENP^OFF^ cells expressing INCENP ST752EE. Arrowheads highlight enrichment of GFP–anillin at the cortex. Scale bars: 10 µm. (G) Upper and middle row, staining of F-actin (red) together with DNA (DAPI, blue) on colcemid-treated INCENP^OFF^ cells expressing INCENP ST752EE. Lower row, GFP–RhoA (GFP:RhoA) together with DNA (DAPI, blue) labeling of INCENP^OFF^ interphase cells expressing INCENP ST752EE. Arrowheads highlight enrichment of F-actin and GFP–RhoA at constriction sites. Scale bars: 5 µm.

Live and fixed-cell analysis of INCENP^OFF^ cells expressing INCENP^ST752EE^ revealed a remarkable increase in plasma membrane blebbing, with ectopic furrows forming in both interphase and mitotic cells (Fig. 5). Substantial plasma membrane contractions were observed in mitosis as early as prophase (Fig. 5Ab) and, remarkably, even in interphase cells (Fig. 5Aa). Ectopic contractile furrows were also observed during cytokinesis (Fig. 5Ac). Although normal in many other respects (see below), these ectopic furrows lacked normal cortical localization of INCENP.

Using time-lapse video microscopy to track and evaluate membrane blebbing and ectopic furrowing events, we measured two different parameters: the time spent in contraction expressed as a percentage of the total movie time and the percentage of elongation that cells undergo from their resting state (Fig. 5C and Fig. S5A, respectively). We obtained a strong correlation between these two methods (σ^2^=0.91) suggesting that both parameters are accurate monitors of blebbing/furrowing events. This correlation is consistent with early reports showing that equatorial contraction is accompanied by polar relaxation resulting in cell elongation (Bray and White, 1988).

INCENP^OFF^ cells expressing either INCENP^T753E^ or INCENP^ST752EE^ exhibited a remarkable increase in contractility (Fig. 5C,D). For INCENP^ST752EE^, we observed an 8-fold increase in the time spent in contraction (16.8±2.3% of the movie time, compared to 2.1±0.7% in control INCENP^OFF^ cells expressing INCENP^WT^; mean±s.e.m.; Fig. 5C). We also measured a 3-fold increase in the degree of elongation (average of 45.5±4.4% compared to 16.9±2.1% in control cells; Fig. S5A). These values for INCENP^OFF^ cells expressing INCENP^ST752EE^ rose dramatically to 55.8±5.8% time in contraction and a 100.3±6.3% degree of elongation in the presence of colcemid (Fig. 5B; Fig. S5A). Thus, cells display enhanced contractility in the absence of a microtubule network. A similar increase in contractility had previously been reported for KE37 lymphoblastoid cells following colcemid treatment (Bornens et al., 1989).

Cytokinetic furrowing is a classical cell cycle-coupled event, with Aurora B, Plk1 and other proteins required for cytokinesis typically accumulating in G2. We therefore determined whether the ectopic furrows triggered by INCENP STD motif mutants occur preferentially in G2 or also in other cell cycle phases. INCENP^OFF^ cells expressing INCENP^ST752EE^ were treated with BrdU in order to label S phase and G2 and M phase cells. Brief colcemid treatment avoided slippage of BrdU-positive cells out of mitosis (Fig. S5B). Expression of the mutant protein had no detectable effect on cell cycle progression (i.e. the percentage of the culture in S phase). A similar frequency of ectopic furrowing events was observed in BrdU negative (G1) and positive (S+G2+M) cells (Fig. 5E). Thus, furrowing induced by INCENP^ST752EE^ can occur regardless of the cell cycle stage.

### INCENP^ST752EE^-dependent ectopic furrows partly resemble cytokinetic furrows

In order to further characterize the ectopic furrows, we investigated the localization of several proteins required for contractile ring formation. We observed prominent F-actin rings in both interphase and mitotic cells undergoing INCENP^ST752EE^-induced furrowing (Fig. 5G). We also observed an enrichment of both exogenously expressed GFP–anillin and GFP–RhoA at constriction sites and at the cortex in INCENP^OFF^ cells expressing INCENP^ST752EE^ (Fig. 5F,G). This contrasts with the normal localization of anillin, which is localized within the nucleus of interphase cells (Field and Alberts, 1995).

Ectopic furrows almost always appeared in the vicinity of chromosome clusters in mitotic cells or close to the nucleus in interphase cells (Fig. 5; Fig. S5). This suggests that the contractile signal may be initiated on or near chromatin and then transmitted to the cell cortex. However, we cannot at this point determine whether furrowing is triggered by local action of low levels of membrane-associated CPC containing INCENP^ST752EE^, or whether the CPC activates a signaling pathway that acts at a distance.

The ectopic furrows exhibit at least two important differences from classical cytokinetic furrows. INCENP and the other members of the CPC normally localize to the equatorial cortex, even before furrowing (Earnshaw and Cooke, 1991; Eckley et al., 1997). In contrast, the INCENP mutant protein was not detectable at the ectopic furrows despite localizing normally to the midbody in dividing cells (Fig. 5Ac).

Secondly, the ectopic furrows do not seem to be associated with PRC1 (Fig. S5C). This can be explained by their lack of association with a central spindle. Together, the lack of INCENP and PRC1 at ectopic furrows suggests that they may frequently fail to complete abscission, as both PRC1 and the CPC have been reported to be important regulators of this process (Carmena et al., 2012; Hu et al., 2012). We note, however, that we have observed ‘cells’ lacking any detectible DNA in these cultures, suggesting that the ectopic furrows occasionally lead to abscission despite the absence of key proteins.

Taken together, our data indicate that mimicking constitutive phosphorylation on S752 and T753 in the INCENP STD motif triggers a potent contractile signal uncoupled from normal cell cycle controls. We therefore set out to further characterize this signaling pathway.

### Plk1 specifically phosphorylates INCENP T753 after priming on S752 *in vitro*

**Fig. 6.**
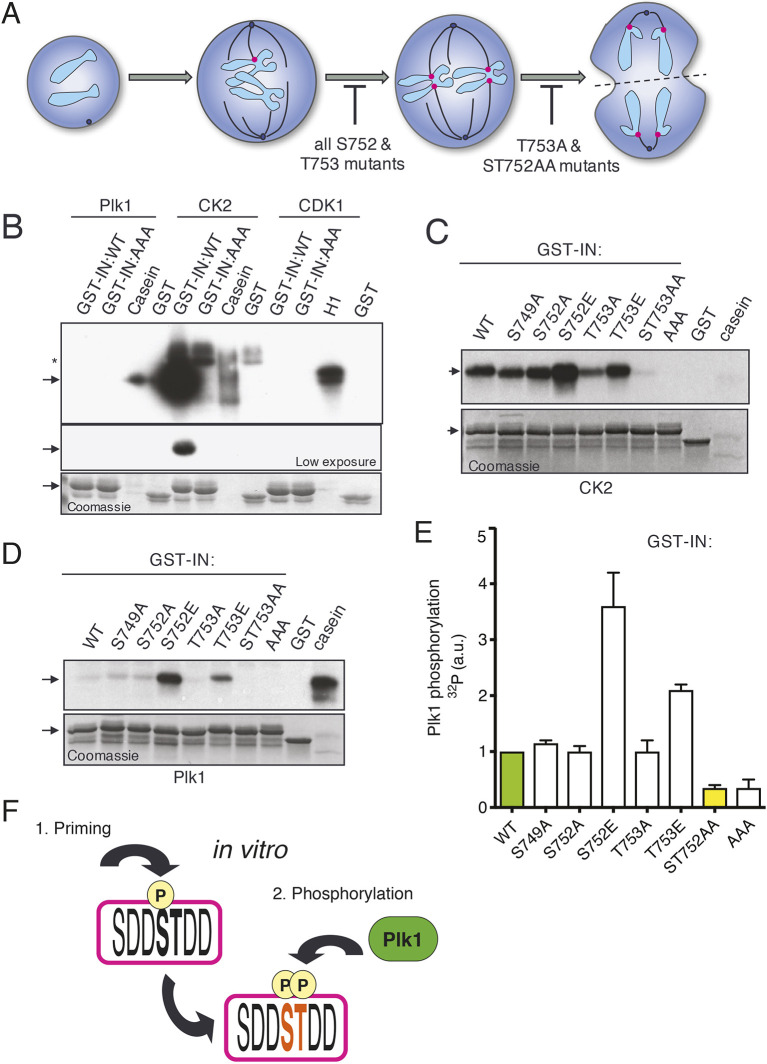
**Plk1 specifically phosphorylates Thr753 after priming on Ser752 *in vitro*.** (A) Diagram showing effects of INCENP mutations on S752 or T753 or both on chromosome alignment or cytokinesis. (B) GST–*Gg*INCENP^737-839^ wild type (GST-IN:WT), GST–*Gg*INCENP^737-839^ bearing a triple mutation S749A–S752A–T753A (GST-IN:AAA), casein or histone H1 were used as substrates in kinase assays for PLK1, CK2 and CDK1. A lower exposure is shown and the star indicates auto-phosphorylated CK2. The Coomassie gel staining shows protein loading. (C) GST–*Gg*INCENP^737-839^ wild type or GST–*Gg*INCENP^737-839^ bearing different mutations on the SDDSTDD motif were used as substrates in kinase assays for CK2. The Coomassie gel staining shows protein loading. (D) Phosphorylation of GST–*Gg*INCENP^737-839^ wild type by PLK1 compared with GST–INCENP^737-839^ bearing different mutation on the SDDSTDD motif. Arrows in B–D highlight GST–*Gg*INCENP^737-839^ polypeptide. (E) ^32^P signal quantification of two independent PLK1 kinase assay experiments such as in D, *n*=2 independent experiments. Values are means±s.e.m. (F) Model showing the priming event on S752 allowing PLK1 phosphorylation on the T753 of *Gg*INCENP.

Because phosphorylation of the INCENP STD motif appears to be required both for accurate chromosome alignment and for cytokinesis (Fig. 6A), we undertook a candidate approach in order to uncover upstream kinase(s) capable of phosphorylating this motif. S752 and T753 are located within putative Plk1 and casein kinase 2 (CK2) consensus sites (D/E-X-S/T-phi-X-D/E and S/T-X-X-D/E/pS, respectively) (Meggio et al., 1994; Nakajima et al., 2003), prompting us to investigate whether either of these kinases could phosphorylate the C-terminal domain of INCENP (aa 737–839) fused to GST. CDK1 was used as a negative kinase control and the triple non-phosphorylatable mutation of the STD motif (S749A, S752A and T753A) as a negative substrate control. We refer to these substrates as GST-IN:WT and GST-IN:AAA, respectively.

GST-IN:WT was very efficiently phosphorylated by CK2 (Fig. 6B) whereas GST-IN:AAA was not. Thus, the STD motif can be specifically phosphorylated by CK2. In contrast, neither Plk1 nor CDK1 phosphorylated GST-IN:WT to detectable levels (Fig. 6B). Looking at single mutants of the STD motif in CK2 kinase assays, we observed a stronger phosphorylation signal on the phosphomimic mutant of S752 (GST-IN:S752E) when compared to phosphorylation of the wild-type peptide (Fig. 6C). Building on this result, and since we previously identified a synergistic effect between S752 and T753 phosphorylation in rescuing cytokinesis (Fig. 4B), we next tested whether mimicking phosphorylation on either S752 or T753 could act as a priming event for Plk1 phosphoryation of the GST–C-term-INCENP peptide. Indeed, mimicking phosphorylation on S752 (GST-IN:S752E) strongly stimulated phosphorylation of the GST:C-term-INCENP peptide by Plk1 *in vitro* (Fig. 6D). Mimicking phosphorylation of T753 (GST-IN:T753E) also stimulated Plk1 phosphorylation of the peptide, although to a lesser extent (Fig. 6D).

Further support for the importance of the highly anionic STD motif in normal mitosis was obtained by mutating D751 or D755 to arginine. This renders the region less acidic and disrupts the Plk1 consensus site. Cells expressing INCENP^D751R^ or INCENP^D755R^ exhibited phenotypes similar to those seen in INCENP^OFF^ cells (Fig. S6A). In both cases, we observed a substantial increase in multinucleated cells, indicative of cytokinesis failure (64.5% for INCENP^OFF^ cells compared to 59.3% for INCENP^OFF^ cells expressing INCENP^D751R^ and 71.6% for INCENP^OFF^ cells expressing INCENP^D755R^; Fig. S6B).

We have not been able to prepare a phospho-specific antibody recognizing various partly phosphorylated forms of the STD motif (0/16 rabbits). It was technically not possible to synthesize the fully phosphorylated peptide and the highly charged doubly phosphorylated peptides were, as predicted by the providers, non-antigenic. However, as described above, both our own study and numerous other studies have confirmed that these residues on INCENP are phosphorylated during mitosis (Nousiainen et al., 2006; Dephoure et al., 2008; Malik et al., 2009; Ohta et al., 2016) and two studies have implicated Plk1 as being involved in that phosphorylation (Hegemann et al., 2011; Santamaria et al., 2011).

Overall, our results suggest a mechanism in which priming-dependent phosphorylation on S752 leads to Plk1 phosphorylation of T753 in the INCENP STD motif (Fig. 6E,F).

### Ectopic contractile furrows induced by INCENP^ST752EE^ require Plk1 and ROCK1 activation

The ectopic furrowing pathway triggered by expression of INCENP^ST752EE^ is strongly dependent on active ROCK1 and Plk1. We used small-molecule inhibitors to specifically inhibit Aurora B, Plk1, ROCK1, CDK1 and CK2 (ZM447439, GW843682X, Y27632, roscovitine and TBCA, respectively) as well as several downstream effectors in cells expressing INCENP^ST752EE^, and then examined the frequency of furrowing in live cells and in fixed images.

**Fig. 7.**
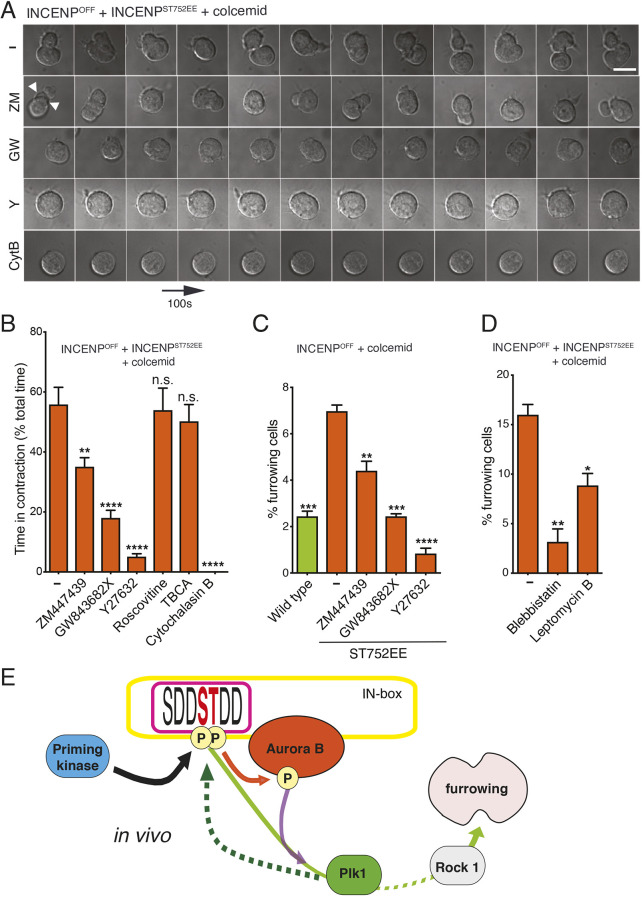
**Ectopic cleavage furrows generated by ST752EE mutation are Plk1-, ROCK1- and Aurora B-dependent.** (A) Frames, 100 s apart from time-lapse live-cell DIC imaging of INCENP^OFF^ cells expressing INCENP ST752EE in interphase treated or not (–) with ZM447439 (ZM), GW843682X (GW), Y27632 (Y) or Cytochalasin B (CytB) in the presence of colcemid. Scale bar: 10 µm. (B) Measurement of time in contraction from DIC movies (as a percentage of total movie time) in INCENP^OFF^ cells expressing ST752EE mutation in the presence of colcemid treated or not with ZM447439, GW843682X, Y27632, Roscovitine, TBCA or cytochalasin B; from 17 to 40 cells per condition were analyzed. (C) Quantification of furrowing events on fixed sample of INCENP^OFF^ colcemid-treated cells expressing INCENP wild-type compared with INCENP^OFF^ colcemid-treated cells expressing the ST752EE mutation treated with ZM447439, GW843682X or Y27632, *n*=3 independent experiments. (D) Quantification of furrowing events on fixed sample of INCENP^OFF^ colcemid-treated cells expressing ST752EE mutation treated with Blebbistatin or Leptomycin B, *n*=3 independent experiments. Results are mean±s.e.m. *P≤0.05; **P≤0.01; ***P≤0.001; ****P≤0.0001; n.s., not significant, *P*>0.05 (two-tailed unpaired *t*-test).

Analysis of the live-cell imaging revealed that the ROCK1 pathway was essential for furrowing (Movies 7 and 10). Treatment with ROCK1 inhibitor Y27632 led to a 91% decrease in the time spent in contraction (Fig. 7A,B). We also observed a substantial inhibition of the maximum elongation upon Y27632 treatment (74% decrease; Fig. S6D). These observations were confirmed by measurements of fixed cells, in which the percentage of furrowing cells was decreased by 88% (Fig. 7C).

This analysis also revealed a critical role for Plk1 in the ectopic furrowing pathway induced by INCENP^ST752EE^. Plk1 inhibition with GW843682X reduced the percentage of furrowing cells to the level observed in colcemid-treated INCENP^OFF^ cells expressing wild-type INCENP (a 64% reduction; Fig. 7C; Movies 7 and 9). As with the treatment with ROCK1 inhibitor, treatment with the Plk1 inhibitor also caused a highly significant decrease in the time spent in contraction (68% decrease; Fig. 7B) and in the maximum elongation (44% decrease; Fig. S6C,D), as scored in the live-cell analysis.

Aurora B also plays a role in the ectopic furrowing. Although inhibition of this kinase did not have as dramatic an effect as the inhibition of ROCK1 or Plk1, addition of ZM447439 led to a 36% reduction in the percentage of furrowing cells and a 37% decrease in the time spent in contraction (Fig. 7B,C; Movies 7 and 8). This suggests that the ectopic signaling pathway must at least partly involve CPC activity (Fig. 7A–C; Fig. S6D).

CDK1, a key kinase implicated in early mitotic events is apparently not involved in this pathway, as its inhibitor roscovitine had no inhibitory effect on membrane contractility. CK2, is also apparently not essential for this pathway, as TBCA also had no inhibitory effect on membrane contractility (Fig. 7B; Fig. S6D). Thus, another kinase must be capable of priming phosphorylation of the STD motif to promote Plk1 phosphorylation.

As expected, the actin-depolymerizing drug cytochalasin B totally abolished furrowing events (Fig. 7A,B; Movie 11). Blebbistatin, a drug affecting myosin 2 ATPase activity (Straight et al., 2003), reduced ectopic furrowing by 80% compared to untreated cells, thus confirming the role of myosin II in ectopic furrow ingression (Fig. 7D). Indirect evidence supporting the involvement of anillin in the ectopic furrowing events observed during interphase is provided by the moderate (45%) inhibition of furrowing seen in the presence of the nuclear export inhibitor leptomycin B (Fig. 7D). Anillin is reported to normally be nuclear in interphase (Chen et al., 2015), but we observed cortical anillin in cells undergoing ectopic furrowing (Fig. 5F).

Taken together, these results suggest that phosphorylation of the INCENP STD motif creates a highly anionic patch that triggers a furrowing initiation pathway that is dependent on Plk1 activity and can be uncoupled from normal cell cycle controls. Ectopic furrowing induced by STD motif mutations shares many characteristics with the formation of normal cleavage furrows seen during mitotic exit.

## DISCUSSION

Cytokinesis is spatially and temporally coordinated with chromosomal events to ensure accurate chromosome segregation (D'Avino et al., 2015; Pollard and O'Shaughnessy, 2019). This coordination is achieved at least partly through the actions of Plk1 and Aurora B kinase in the chromosomal passenger complex (CPC). Aurora B and Plk1 work together in cytokinesis to release scaffolding proteins from the central spindle, to allow them to oligomerize and to recruit the RhoA GEF Ect2 to the plasma membrane where it initiates the assembly of the contractile apparatus (Su et al., 2011; Kotynkova et al., 2016; Adriaans et al., 2019).

Here, we describe a highly conserved phosphorylated region of the CPC scaffolding protein INCENP that regulates Aurora B activity and, when mutated to mimic constitutive phosphorylation, initiates a dominant furrowing signal that is uncoupled from cell cycle controls. Our results suggest that controlled phosphorylation of this region is required for chromosome alignment and full SAC activity in early mitosis. They also suggest a novel switch mechanism in which sustained INCENP phosphorylation by Plk1 at anaphase onset may trigger furrowing initiation. Thus, the CPC may act earlier in triggering furrowing than has previously been appreciated.

### The STD motif regulates Aurora B activity and cytokinesis

The IN-Box is a short stretch of homology between INCENP proteins in vertebrates, *Drosophila*, *C. elegans* and yeasts (Adams et al., 2000). The most highly conserved region of the INCENP polypeptide lies near the N-terminal end of the IN-Box and is extremely rich in serine, threonine and aspartate residues. We therefore term this region the STD motif. Crystal structures reveal that the IN-box drapes around the small lobe of Aurora B kinase like a crown (Sessa et al., 2005; Elkins et al., 2012). At the other end of the IN-box from the STD motif are the TSS residues whose phosphorylation plays a key role in Aurora B activation (Schumacher et al., 1998).

Coincidentally, the shorter version of the IN-Box used in structural studies just misses the STD motif, beginning with its C-terminal aspartate (Sessa et al., 2005; Elkins et al., 2012). Examination of those crystal structures suggests that the STD motif is located in the vicinity of the catalytic cleft of Aurora B. This might explain why STD motif mutants negatively impact on H3S10 phosphorylation by the kinase. This highly negative region could modulate the structure of the catalytic cleft or it could influence the binding or release of highly positive substrates such as the histone N-terminal tails. Phosphorylation of the STD motif appears to reflect a previously undescribed mechanism for regulating Aurora B activity.

Proper phosphorylation of the STD motif is essential for mitotic regulation. Mutations of S752 or T753 to an alanine have the strongest effects, failing to rescue both chromosome alignment and cytokinesis. Expression of phosphomimetic mutants of these residues gives more-complex results, apparently separating INCENP functions in chromosome alignment from those in cytokinesis. Single and double mutations of S752 or T753 to glutamate all fail at chromosome alignment but are not significantly different from wild-type INCENP at rescuing cytokinesis in INCENP^OFF^ cells.

### Phosphomimetic mutations of the STD motif uncouple membrane furrowing from cell cycle regulation

Phosphomimetic mutations of the STD motif have an unexpected and remarkable phenotype – they trigger furrowing in cells independently of the cell cycle and of microtubules. Furrowing can occur in G1 or G2 phases or even in mitosis in the presence of colcemid. The fact that the furrowing can occur in G1 phase reveals that the INCENP^ST752EE^ pathway can be activated at times in the cell cycle when several factors thought to be involved in mitotic regulation are present either at low levels or in an inactive form. Nonetheless, the process appears to closely resemble early stages of normal cytokinesis.

Although surprising, such an interphase furrowing pathway is not without precedent. Interphase cortical furrowing stimulated by microtubule disassembly was previously reported in the human KE37 lymphoblastoid cell line, though the molecular mechanism was not determined (Bornens et al., 1989). Overexpression of the fission yeast Plk1 ortholog Plo1, leads to ectopic septum formation in interphase G1 and G2 *S. pombe* cells (Ohkura et al., 1995). In *Drosophila* unfertilized eggs, injection of the CDK1 inhibitor RO3306 or a constitutively active RhoA at the cortex induces furrowing (Menant and Karess, 2012). In a more recent study, RhoA activation at the cell cortex by optogenetic targeting of the GEF domain of the LARG protein induced ectopic furrowing in interphase cells and mitotic cells in the presence of nocodazole (Wagner and Glotzer, 2016). These furrows were sustained only in the presence of the activating light and reversed shortly after it was extinguished. As observed here for INCENP^ST752EE^-expressing cells, the furrows did not typically go to completion.

Taken together, these results confirm that furrow initiation does not require components expressed or activated only during mitotic exit.

### Plk1 phosphorylates the STD motif

The fact that phosphomimetic mutations of the STD motif trigger furrowing strongly suggests that kinase(s) that phosphorylate this motif may control furrow initiation. We show here that a phosphomimetic mutant of INCENP S752 activates T753, which is a Plk1 phosphorylation target *in vitro*. Indeed, there is now considerable evidence linking Plk1 to control of furrow initiation. *S. pombe* Plo1 is essential for septation (Ohkura et al., 1995) and *Drosophila* Polo kinase is required for cytokinesis (Carmena et al., 1998). More recently, Plk1 has been shown to be essential for the initiation of cytokinesis in mammalian cells (Burkard et al., 2007; Petronczki et al., 2007, 2008), possibly acting in more than one parallel pathway (Lewellyn et al., 2011; Adriaans et al., 2019).

The INCENP STD motif is a highly conserved putative Plk1 consensus site. Previous phosphorylation analysis of human mitotic protein complexes found that phosphorylation of *Hs*INCENP S828, S831 and T832 (corresponding to *Gg*INCENP S749, S752 and T753) is sensitive to the specific Plk1 inhibitor BI4834 (Hegemann et al., 2011). In addition, S831 and T832 phosphorylation was downregulated in prometaphase cells either depleted of Plk1 or treated with the Plk1 inhibitor TAL (Santamaria et al., 2011).

Sequence alignment of the STD motif shows that in yeasts, only the serine (S752 equivalent) is phosphorylatable. The T753 equivalent is replaced by glutamate or asparate in budding and fission yeasts, respectively. Nonetheless, we see the most severe effects on both chromosome alignment and cytokinesis in DT40 cells when INCENP is mutated on T753. Importantly, the overall negative charge of the domain is conserved in yeasts, suggesting that it is likely involved in electrostatic interactions related to a conserved function. This could include acting as a priming site for yeast Plk1 (Cdc5 and Plo1). Thus, there may be an additional level of regulation in vertebrates compared to yeast.

The priming kinase that phosphorylates INCENP on S752 *in vivo* is unknown. However, CK2 can phosphorylate INCENP *in vitro* on both S752 and T753. CK2 was reported to be involved in early mitosis (St-Denis et al., 2009) and it could phosphorylate INCENP, priming it for subsequent Plk1 phosphorylation.

Alternatively, Plk1 may act as its own priming kinase for S752 and T753. Phosphorylation of both homologous residues in human INCENP is sensitive to Plk1 inhibition or depletion (Hegemann et al., 2011; Santamaria et al., 2011). Furthermore, we found that phosphorylation of S752 can prime Plk1-dependent phosphorylation on T753, and vice versa, although to a lesser extent. Together, these findings could point towards a feed-forward activation loop between the two sites upon Plk1 phosphorylation.

Whatever the mechanism of Plk1 activation, the kinase must act at multiple steps. Firstly, it phosphorylates the STD motif, and this is essential for both chromosome alignment and cytokinesis. Secondly, it must act again downstream of the phosphorylated STD motif, since furrowing induced by expression of INCENP^ST752EE^ was strongly inhibited following Plk1 inhibition with GW843682X.

### Control of the ectopic furrowing pathway

Although ectopic furrows induced by INCENP^ST752EE^ share numerous features with normal contractile furrows, including the presence of RhoA, cortical anillin and an actin–myosin contractile ring, remarkably, furrowing occurs without local INCENP accumulation. Indeed, INCENP localization was known to be dispensable for the initiation of furrowing and furrow positioning (Mackay et al., 1998; Shannon et al., 2005). Current data do not rule out a mechanism in which INCENP and the CPC act at a distance to release a signal that initiates the furrowing pathway. The lack of a local concentration of factors originating from a spindle midzone, such as INCENP (Cooke et al., 1987; Earnshaw and Cooke, 1991) and PRC1 (Mollinari et al., 2002), could explain why furrows in cells expressing INCENP^ST752EE^ do not typically complete abscission.

Importantly, the furrowing signal from INCENP^ST752EE^ is apparently upstream of Ect2 recruitment and RhoA activation at the plasma membrane. Optogenetic activation of RhoA at the membrane is insensitive to Plk1 inhibitors (Wagner and Glotzer, 2016), whereas the INCENP^ST752EE^ pathway is blocked by inhibition of Plk1 and, more weakly, Aurora B. The latter finding suggests that Aurora B facilitates but is not essential for this signaling pathway. Indeed, phosphomimetic STD motif mutations diminish Aurora B activity by ∼50% in prometaphase cells. Thus, the novel dominant furrow-initiation pathway described here does not involve hyperactivation of Aurora B and indeed ectopic furrowing was not observed when Aurora B was overexpressed in a mouse model (González-Loyola et al., 2015).

In the normal mitotic furrowing pathway, Plk1 phosphorylation of Cyk4 creates a docking site that recruits Ect2 in the anaphase spindle midzone (Burkard et al., 2009). Plk1 then releases the complex from PRC1 (Adriaans et al., 2019) and Aurora B kinase allows its oligomerization and association with the cortex, where it can activate RhoA in a microtubule-independent manner (Basant et al., 2015). Indeed, furrowing during interphase is actually promoted by microtubule disassembly as shown here and in KE37 cells (Bornens et al., 1989). Furthermore, PRC1 regulation is unlikely to be involved in the interphase furrowing pathway, as PRC1 appears to be nuclear and is specifically expressed and activated in mitosis (Jiang et al., 1998; Mollinari et al., 2002). It is possible, however that in the presence of constitutive INCENP expression, Aurora B might function throughout the cell cycle in regulating Cyk4 oligomerization.

How the phosphorylated STD motif promotes furrowing requires further study. This extremely anionic patch could bind to a positively charged ligand to target the CPC to specific substrates or to modulate the kinase activity towards particular substrates. If phosphorylation of the patch regulates CPC binding to histones, this might help to explain the delay in INCENP^ST752EE^ transfer to the spindle at anaphase onset. Whatever the target, the STD motif is critical for INCENP function, since the INCENP^ST752AA^ mutant behaves essentially as INCENP-null both for chromosome biorientation and cytokinesis.

### Conclusion

INCENP is an essential cofactor for Aurora B kinase in the CPC. The analysis of the highly conserved STD motif presented here reveals that INCENP is also both a target of Plk1 and involved in a downstream Plk1-dependent pathway to promote plasma membrane furrowing. Thus, as speculated previously (Carmena and Earnshaw, 2006), INCENP appears to be a central player in coordinating, relaying and tuning essential signals sent by kinases orchestrating the different steps of mitosis.

## MATERIALS AND METHODS

### Cell culture and model

DT40 cells were grown as previously described (Buerstedde and Takeda, 1991). The INCENP conditional knockout cells were described and analyzed in our previous studies (Samejima et al., 2008; Xu et al., 2009). Doxycycline, at a final concentration of 1 µg/ml, was added to the culture medium for a minimum of 24 h to repress transcription of the *INCENP* gene. TK6-CDK1as cells were obtained and synchronized with 1NM-PP1 at 2 µM for 12 h as described previously (Gibcus et al., 2018; Samejima et al., 2018).

### Immunoblotting and antibodies

Whole-cell lysates were prepared by lysing the cells in sample buffer. SDS-PAGE and immunoblotting were performed following standard procedures. Anti-α-tubulin antibody (clone B512, T5168, 1:2000) and anti-H3 phospho-Ser10 (06-570, 1:1000) were purchased from Sigma and Upstate Biotech (now Merck-Millipore), respectively, and anti-Plk1 pT210 from Abcam (ab39068, 1:500). Rabbit polyclonal (WCE1186, 1:500) and mouse monoclonal anti-INCENP (3D3, 1:500) were previously described (Cooke et al., 1987; Mackay et al., 1993). For F-actin staining, phalloidin conjugated to tetramethyl rhodamine B purchased from Sigma was used at a 1:500 dilution. Rabbit anti-PRC1 antibody was raised against full-length His-tagged chicken PRC1 protein and used at 1:500 dilution.

### Indirect immunofluorescence microscopy

Cells were incubated at 39°C on poly-lysine-coated slides (Polysine™ from VWR International) for 10 min before fixation in 4% PFA in PBS buffer at 37°C and permeabilization in 0.15% Triton X-100 in PBS buffer for 2 min. After blocking in 1% BSA in PBS with 0.05% Tween 20 for 1 h, cells were probed with the antibodies described above and slides mounted using Vectashield containing DAPI (Vector laboratories). Image stacks were taken using an Olympus IX-70 microscope with a charge-coupled device camera (CH350; Photometrics) controlled by Delta Vision SoftWorks (Applied Precision) and a 100× objective (NA 1.4). Image stacks were deconvolved using SoftWorks and maximum intensity or sum projections were generated.

For immunofluorescence quantification of H3S10ph, prometaphase cells were imaged and sum projections of equal numbers of stacks were generated. The H3S10ph signal on chromosomes was normalized against the DAPI levels after background deduction and analyzed using ImageJ software. All graphs and data statistical analysis were done using GraphPad Prism 5. Each data set was expressed as the mean±s.e.m. and *P*-values were determined by *t*-test. *P*-values are shown by means of asterisks as follows: n.s., not significant; *P*>0.05; *P≤0.05; **P≤0.01; ***P≤0.001; ****P≤0.0001.

### Live-cell imaging

For live-cell imaging by DIC microscopy, DT40 cells were seeded onto concanavalin A-coated coverslips (0.1 mg/ml) for 30 min. The coverslips were then transferred to a Rose Chamber and kept at 39°C in the presence of RPMI1640 medium plus 10% FBS, without Phenol Red. DIC images were collected every 10 s with a COOLSNAP HQ2 camera on an inverted Nikon Eclipse TE2000-E microscope heated to 39°C controlled by Metamorph (Molecular Devices), using a 100× NA 1.40 objective.

Small-molecule inhibitors were added to the culture 3 h prior to image acquisition and colcemid at 0.1 µg/ml 2 h prior to image acquisition. Concentrations used were as follows: Aurora B inhibitor, ZM447439 at 4 µM; Plk1 inhibitor, GW843682X at 1 µM; ROCK1 inhibitor, Y27632 at 4 µM; CDK1 inhibitor, roscovitine at 10 µM; and CK2 inhibitor, TBCA at 10 µM. Cytochalasin B was used at 40 µM, Blebbistatin at 50 µM and Leptomicin B at 130 nM.

For fluorescence live-cell imaging, INCENP-knockout cells and mutant rescue cells stably expressing H2B–RFP were transfected with a targeting construct in order to insert GFP downstream of the CENP-H gene by homologous recombination as previously described (Vagnarelli et al., 2006). The CENP-H–GFP knock-in construct was provided by Tatsuo Fukagawa (Graduate School of Frontier Biosciences, Osaka, Japan). Fluorescence images were collected on an Olympus IX-70 microscope controlled by Delta Vision SoftWorx (Applied Precision) and a 100× objective (NA 1.4) every 2 min prior to anaphase onset then, three-dimensional data sets were collected every minute onwards. Movie frames were processed using Delta Vision SoftWorx software (Applied Precision).

### Site-directed mutagenesis

INCENP point mutants were generated by site-directed mutagenesis (QuikChange™ site-directed mutagenesis kit from Stratagene) using pZeo *Gg*INCENP class I vector (previously reported in Samejima et al., 2008; Xu et al., 2009) as template. The different constructs were transfected into the INCENP conditional knockout cells by electroporation. We selected stable clones based on their expression levels of exogenous INCENP being as close as possible to the endogenous protein levels. Clones bearing the S752E,T753E mutation were unstable, with decreasing expression level of the mutant protein over passages and increased cell death even in the presence of the endogenous INCENP protein suggesting a dominant-negative effect.

### Kinase assays and pull-down experiments

Recombinant baculovirus coding for His-tagged Plk1 was generated using Bac-to-Bac system (Invitrogen) and used to infect Sf-9 insect cells. After 48 h, infected cells were pelleted and lysed in lysis buffer (50 mM Tris-HCl pH 8.0, 0.2 M NaCl, 0.5% NP-40, 0.25% deoxycholate, 1 mM PMSF, protease inhibitors, 20 mM β-glycerophosphate and 0.3 mM sodium vanadate), followed by a short sonication then centrifugation (14,000 ***g*** for 30 min). The clear lysate was incubated with Ni-NTA-agarose beads (Qiagen) in presence of 10 mM imidazole for 1 h at 4°C. Beads were washed twice with lysis buffer supplemented with 15 mM imidazole, once with 50 mM Tris-HCl pH 8.0, 0.2 M NaCl, 15 mM imidazole, 0.1% NP-40, 1 mM PMSF, and once in 10 mM Tris-HCl pH 8.0, 15 mM imidazole. The kinase was eluted by incubation of the beads in 250 mM imidazole, 10 mM Tris-HCl, pH7.5 and 150 mM NaCl then dialyzed against 10 mM Tris-HCl pH 7.5, 100 mM NaCl, 0.5 mM EDTA, 1 mM DTT and 0.1 mM PMSF before being analyzed by immunoblotting and used for kinase assays. CK2 and Cdc2/CDK1 were purchased from New England Biolabs. Kinase assays were performed by adding the recombinant kinases to a 20 µl reaction containing the different substrates, 50 mM Tris-HCl pH 7.4, 10 mM MgCl_2_, 1 mM EGTA, 1 mM DTT, 5 mM NaF, 5 mM β-glycerophosphate, 0.05 mM sodium vanadate, 0.1 mM ATP, and 1 µCi of [^32^P]ATP. After 30 min at 30°C, reactions were stopped by the addition of SDS sample buffer. Samples were separated by SDS-PAGE, gels dried and phosphate incorporation determined by PhosphorImager. Signal quantification was performed after measuring ^32^P levels in two independent sets of experiments.

Various mutants of INCENP with an N-terminal Triple Tag (Hexa-His, S, SBP; TrAP) were constructed and stable DT40 INCENP conditional knockout cells expressing these constructs generated. After doxycyclin treatment, cells were lysed using 50 mM Tris-HCl pH 8.0, 250 M NaCl, 1% NP-40, 0.5% sodium deoxycholate, 1 mM PMSF, protease inhibitors, 20 mM β-glycerophosphate, 0.3 mM sodium vanadate and 50 U benzonase, then sonicated. After centrifugation (20,000 ***g***; 15 min, 4°C), lysates were incubated with pre-washed Streptavidin MyOne-C1 Dynabeads for 90 min (Invitrogen). After washing, proteins bound to the beads were separated by SDS-PAGE. For the *in vitro* binding assay, bacterially expressed GST, or wild-type or mutant GST–*Hs*INCENP C-terminus (aa 820–918) bound to glutathione–Sepharose beads, were incubated with lysates from Sf9 infected cells producing His–*Hs*Aurora B (lysis buffer, 50 mM Tris-HCl, pH 8.0, 250 M NaCl, 1% NP-40, 0.5% sodium deoxycholate, 1 mM PMSF, protease inhibitors, 20 mM β-glycerophosphate and 0.3 mM sodium vanadate) (previously reported in Gassmann et al., 2004) for 2 h at 4°C followed by 30 min at room temperature. After washing twice with lysis buffer, once with 50 mM Tris-HCl pH 8.0, 0.2 M NaCl, 0.1% NP-40, 1 mM PMSF and once in 10 mM Tris-HCl pH 8.0, proteins bound to the beads were analyzed by SDS-PAGE and immunoblotting.

### Proteomics of intracellular immunostained subsets

Proteomics of intracellular immunostained subsets, known as PRIMMUS, was performed as follows. Synchronised TK6-CDK1as cells were fixed with 1% formaldehyde for 10 min, quenched with glycine buffer and permeabilized with 90% methanol. Cells were immunostained with anti-H3S28ph (1:200, Abcam, AB_2295065), anti-Cyclin B (1:200, Cell Signaling Technologies, #12231) and anti-Cyclin A (1:200, Cell Signaling Technologies, #4656) antibodies, followed by secondary immunostaining with anti-rabbit-IgG conjugated to Alexa Fluor 647, anti-mouse-IgG conjugated to Alexa Fluor 488 and anti-rat-IgG conjugated to Alexa Fluor 568 (Abcam). Mitotic cells were isolated by sorting cells on a BD Aria Fusion and collecting the H3S28ph+ population. Cells were processed using an ‘in cell digestion’ (T.Y., unpublished observation). Briefly, cells were resuspended in digest buffer (0.1 M triethylammonium bicarbonate, pH 8.5, Sigma-Aldrich), digested with benzonase (Merck Millipore), followed by LysC (Wako), tandem mass tag (TMT) labeling using a 10-plex TMT kit (Thermo Fisher Scientific) and desalted. The desalted peptides were phosphoenriched using Ti:IMAC (Resyn Biosciences) as previously described (Ly et al., 2017). Phosphoenriched peptides were then separated using high pH reverse phase chromatography (Waters BEH 4.6 mm×150 mm C18 column; A, 10 mM ammonium formate, pH 9.0; B, 80% acetonitrile plus 10 mM ammonium formate, pH 9.0) into 16 fractions (Hiraga et al., 2017). Fractions were then dried under vacuum and resuspended in 5% formic acid for liquid chromatography tandem mass spectrometry (LC-MS/MS) analysis.

### LC-MS/MS

LC-MS analysis was performed on an Orbitrap Fusion Lumos Tribrid MS (Thermo Fisher Scientific) coupled on-line, to an Ultimate 3000 RSLCnano HPLC (Dionex, Thermo Fisher Scientific). Peptides were separated on a 50 cm EASY-Spray column (Thermo Fisher Scientific) and ionized using an EASY-Spray source (Thermo Fisher Scientific) operated at a constant temperature of 50°C. Mobile phase A consisted of 0.1% formic acid in water while mobile phase B consisted of 80% acetonitrile and 0.1% formic acid. Peptides were loaded onto the column at a flow rate of 0.3 μl/min and eluted at a flow rate of 0.25 μl/min according to the following gradient: 2 to 40% mobile phase B in 120 min, then to 95% in 11 min. The percentage of mobile phase B remained constant for 10 min and returned to 2% until the end of the run (160 min).

MS1 survey scans were performed at 120,000 resolution (scan range 350–1500 *m*/*z*) with an ion target of 2.0×10^5^ and maximum injection time of 50 ms. MS2 was performed in the ion trap in rapid scan mode with an ion target of 1.0×10^4^ and collision-induced dissociation (CID) fragmentation with normalized collision energy of 35 (arbitrary units) and CID activation time of 10 ms. The quadrupole isolation window was set at 0.7 Th. Only ions with charge between 2 and 7 were selected for MS2. For SPS TMT quantification (McAlister et al., 2014), MS3 scans were performed in the Orbitrap at 60,000 resolution and scan range of 100–150 *m*/*z*. The number of SPS precursors was set to 5 and the isolation window to 2.0 Th. Fragmentation was performed using higher-energy collisional dissociation (HCD) at 65% and the ion target was set to 1.0E5 with a maximum injection time of 120 ms.

### MS data analysis

Raw data files were processed using MaxQuant version 1.6.2.6 (Cox and Mann, 2008), which incorporates the Andromeda search engine (Cox et al., 2011). The spectra were searched against a human FASTA database (accessed June 2018) containing all reviewed entries in the reference UniProt Human Proteome. The processed output was then analyzed using R or RStudio software. TMT reporter ion intensities were corrected for mixing using total ion intensities measured for unmodified peptides.

## Supplementary Material

Supplementary information
