# Supplementary material for: Cell cycle-independent furrowing triggered by phosphomimetic mutations of the INCENP STD motif requires Plk1
Source: J Cell Sci. 2019 Nov 6;132(21):jcs234401. doi: 10.1242/jcs.234401 (PMC7115952; doi:10.1242/jcs.234401)
Supplement: Supplementary information [file joces-132-234401-s1.pdf]

Figure S1 relates to Figure 1. It gives information about MS2 fragmentation spectrum of INCENP phosphopeptides shown in figure 1.

Figure S2 relates to Figure 2. It describes the INCENP conditional knockout model used in the study. It gives information on how phosphomimetic and phosphodeficient mutations on S752 and/or T753 of INCENP affect H3S10 phosphorylation and weaken the checkpoint.

Figure S3 (related to Figure 4) shows how INCENP mutants used in this study transfer to spindle midzone.

Figure S4 (related to Figure 4) focuses on the spindle midzone of the $INCENP^{OFF}$ cells expressing $INCENP^{ST752AA}$ mutant protein.

Figure S5 (relates to Figure 5). It shows the other set of measurements (% of maximum elongation) used to assess furrowing of the $INCENP^{OFF}$ cells expressing $INCENP^{ST752EE}$ mutant protein in presence of colcemid. It describes as well the BrdU experiment in figure 5 and shows PRC1 localization in $INCENP^{OFF}$ cells expressing $INCENP^{ST752EE}$ mutant protein.

Figure S6 (relates to Figures 6,7). It shows that disruption of the Plk1 consensus site leads to an increase of the number of multinucleated cells. It also shows active Plk1 in $INCENP^{OFF}$ cells expressing $INCENP^{ST752EE}$ mutant protein as well as the complementary set of measurements (% of maximum elongation) used to assess furrowing of the $INCENP^{OFF}$ cells expressing $INCENP^{ST752EE}$ mutant protein in presence of various drugs.

Movies 1 to 4 relate to Figure 4C. These are the movies from which the frames were taken.

Movies 5 and 6 relate to Figure S4. They illustrate 3D reconstructions of both $INCENP^{WT}$ and $INCENP^{ST752AA}$ expressing cells in anaphase.

Movies 7 to 11 relate to Figure 7A. These are the movies from which the frames were taken.

Journal of Cell Science • Supplementary information

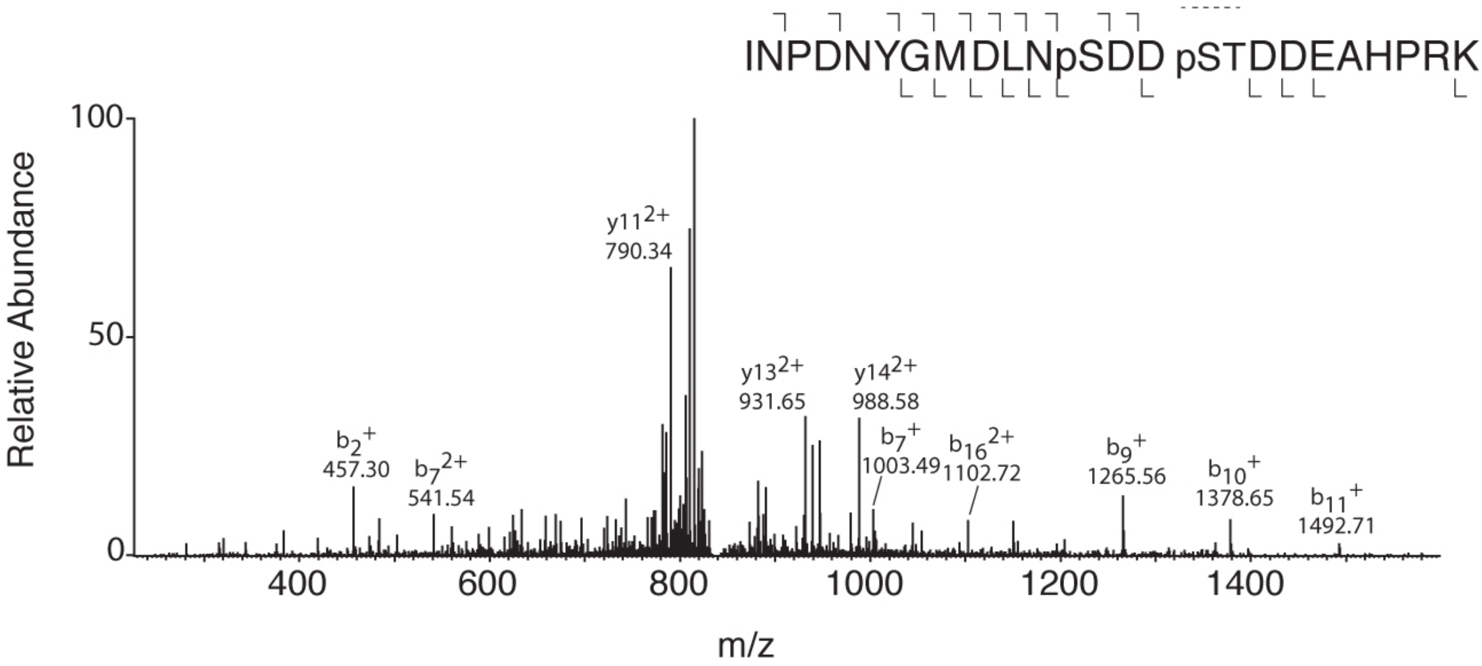

Figure S1: *Example of MS2 fragmentation spectrum of INCENP phosphopeptide.* TMT-labelled, di-phosphorylated INPDNYGMDLNSDDSTDDEAHPRK peptide from INCENP. The inset shows the peptide sequence and the locations of sequence ions (a, b, y, -water, -ammonia). The methionine residue (in italics) is oxidized. The dotted line indicates ambiguity in the location of the second phosphate group (S831/T832). The Andromeda score for this match is 126.

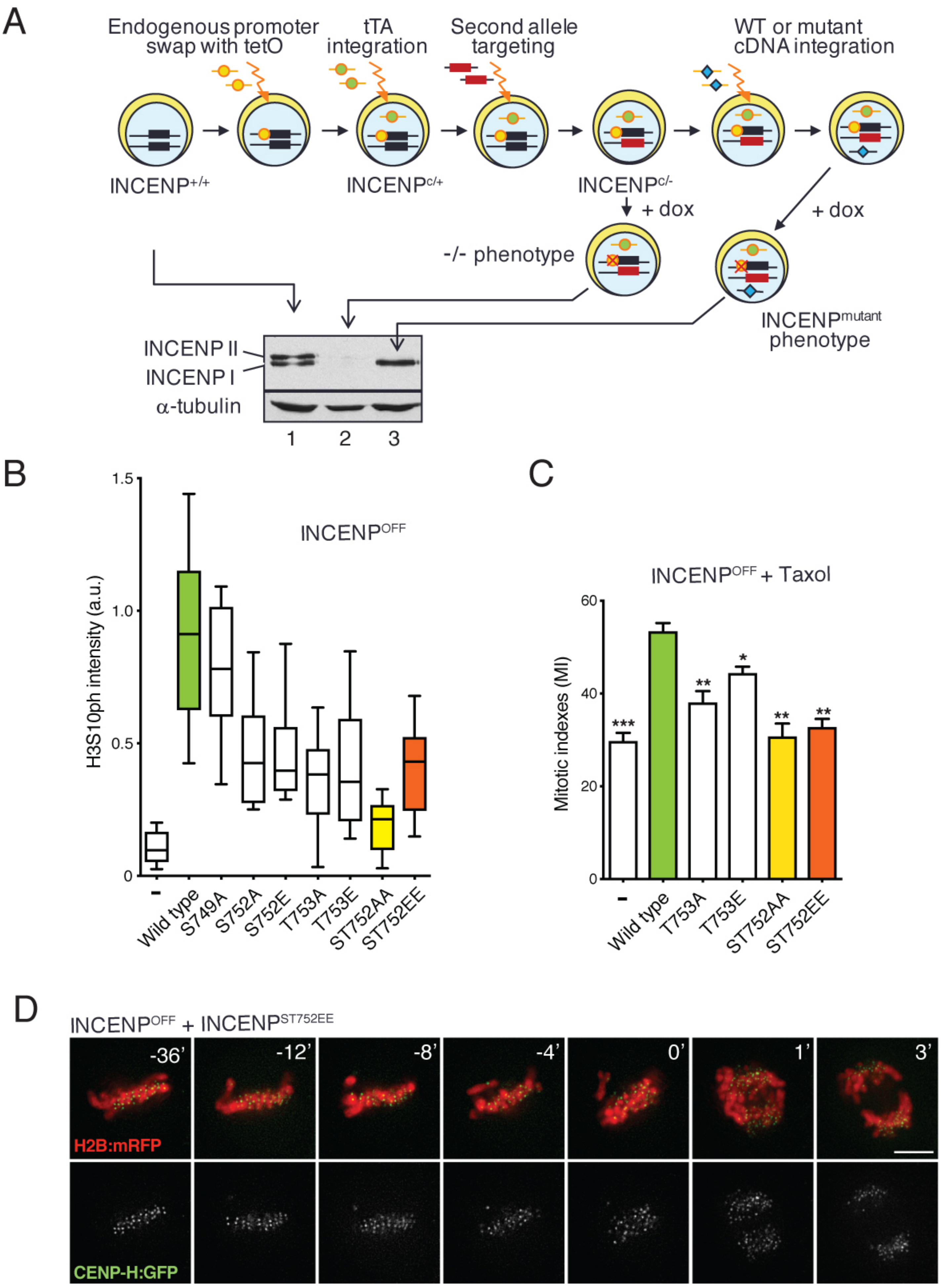
A
Endogenous promoter swap with tetO
tTA integration
Second allele targeting
WT or mutant cDNA integration
INCENP+/+
INCENPc/+
INCENPc/-
+ dox
-/- phenotype
+ dox
INCENPmutant phenotype
INCENP II
INCENP I
α-tubulin
1
2
3
B
INCENPOFF
H3S10ph intensity (a.u.)
1.5
1.0
0.5
0
-
Wild type
S749A
S752A
S752E
T753A
T753E
ST752AA
ST752EE
C
INCENPOFF + Taxol
Mitotic indexes (MI)
60
40
20
0
***
**
*
**
**
-
Wild type
T753A
T753E
ST752AA
ST752EE
D
INCENPOFF + INCENPST752EE
-36’
-12’
-8’
-4’
0’
1’
3’
H2B:mRFP
CENP-H:GFP

Figure S2: *Phosphomimetic and phosphodeficient mutations on S752 and/or T753 of INCENP affect H3S10 phosphorylation and weaken the checkpoint.* **A)** The conditional knockout model of *Gg*INCENP. First, the promoter of the first *INCENP* allele was replaced by the operator Tet (TetO) by homologous recombination. The second step consisted of a random integration of a construction containing the transactivator (tTA) under the control of the promoter of the *INCENP* gene (INCENP$^{c/+}$). In the next step, the promoter of the second *INCENP* allele was replaced by a TetO (INCENP$^{c/-}$). The addition of doxycycline (dox) suppresses the expression of both alleles, thus generating the null phenotype (-/-). Random integration of wild type or mutant *INCENP* cDNAs allowed, after the addition of doxycycline, the observation of a restored wild type phenotype or a mutant phenotype. Western blot is showing *Gg*INCENP protein expression levels in wild cells (INCENP$^{+/+}$), INCENP$^{c/-}$ cells 24 hours after the addition of doxycycline (INCENP^{OFF}) and in stably transfected INCENP^{OFF} cells expressing exogenous *Gg*INCENP class I. Note the presence of two INCENP isoforms, class I and II (INCENP I and II respectively) in wild type cells and the total absence of protein in INCENP^{OFF} cells. **B)** quantification of H3S10ph signal on prometaphase INCENP^{OFF} cells and INCENP^{OFF} cells expressing wild type or various mutants on the SDDSTDD motif of INCENP protein (n>12). **C)** scoring of mitotic INCENP^{OFF} cells, treated for 6 h in 20 nM taxol, expressing the indicated wild type or mutant INCENP proteins. n=3 independent experiments, unpaired t-test, values are means ± SEM, two tailed P-values are indicated with asterisks, n.s.: not significant. **D)** chosen frames from time lapse live cell imaging of INCENP^{OFF} cells expressing ST752EE INCENP mutant protein stably expressing H2B:mRFP (red) and CENP-H:GFP (green). CENP-H:GFP signal (lower frames) and merged are shown. Scale bar: 5 µm.

Journal of Cell Science • Supplementary information

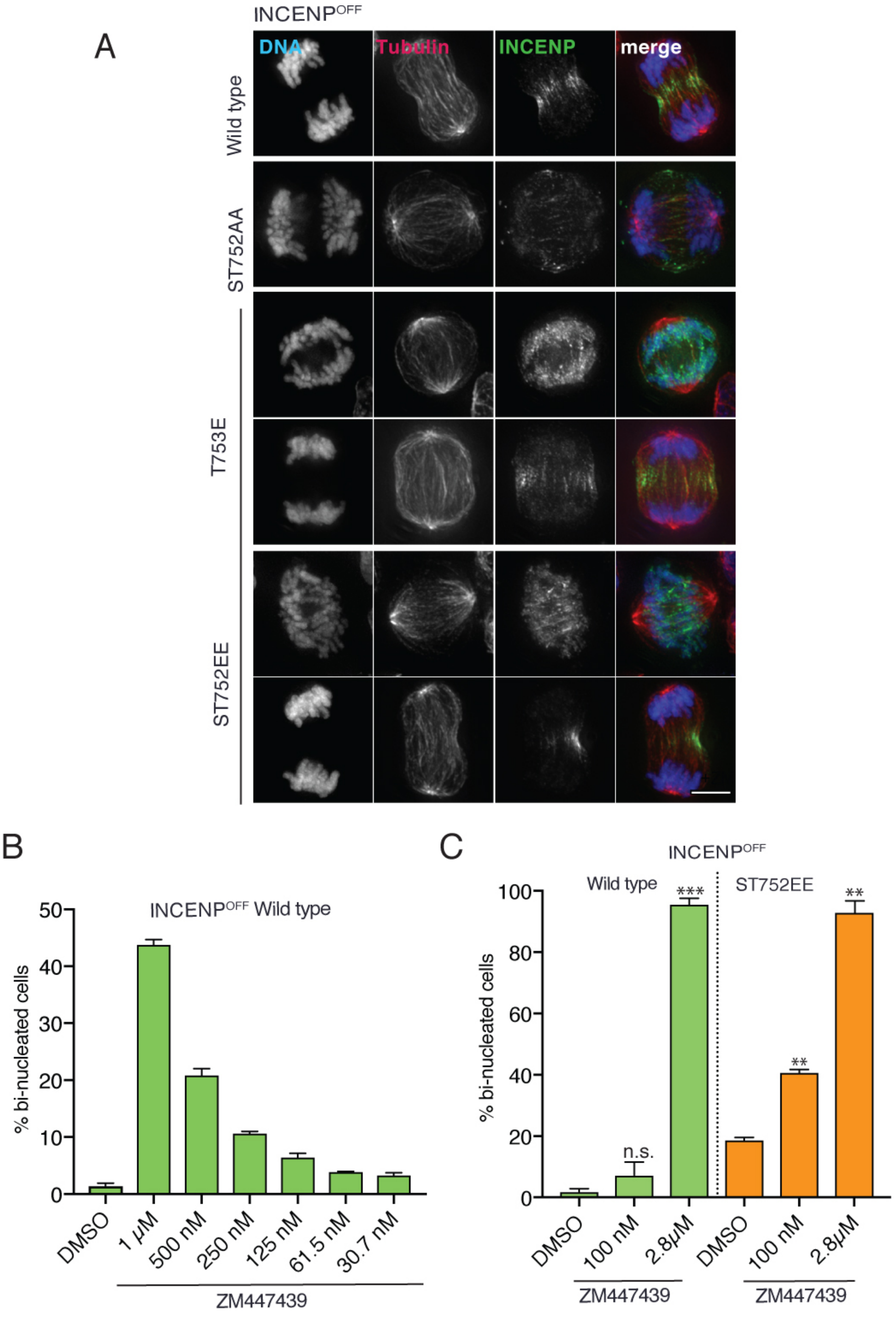

Journal of Cell Science • Supplementary information

Figure S3: *Mutant INCENP proteins localization in anaphase cells.* **A)** immunostaining of INCENP (green) and α-tubulin (red) together with DNA (DAPI, blue) on $INCENP^{OFF}$ cells expressing wild type, ST752AA, T753E and ST752EE mutant INCENP proteins. Scale bar: 5 µm. **B)** quantitation of bi-nucleated cells in $INCENP^{OFF}$ cells expressing wild type INCENP treated with increasing concentrations of the Aurora B inhibitor ZM447439. n=2 independent experiments. **C)** quantitation of bi-nucleated cells in $INCENP^{OFF}$ cells, expressing either wild type or ST752EE INCENP protein, treated with 100 nM or 2.8 µM of ZM447439. n=2 independent experiments, unpaired t-test, values are means ± SEM, two tailed P-values indicated with asterisks, n.s.: not significant.

Journal of Cell Science • Supplementary information

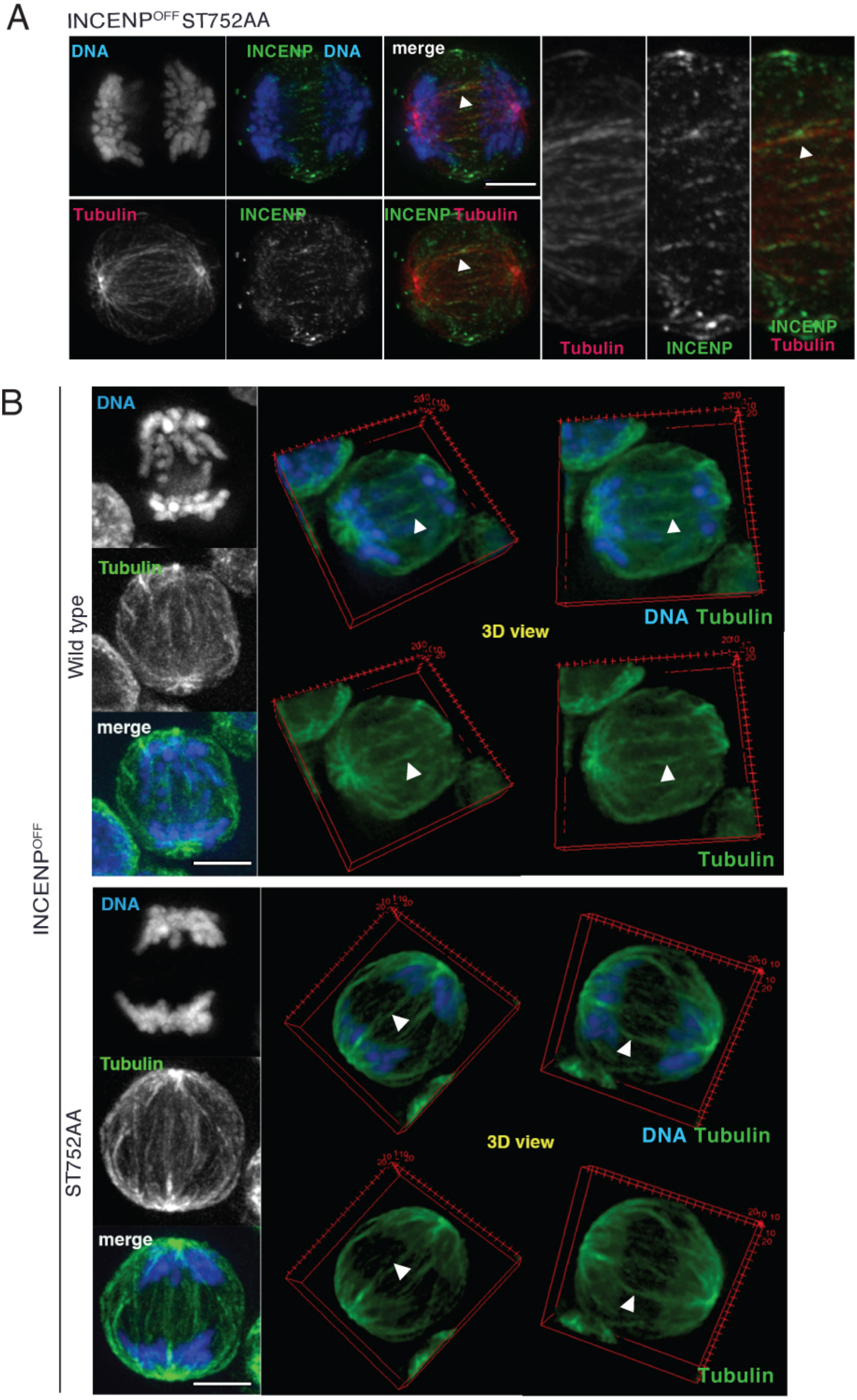
A
INCENP^OFF ST752AA
DNA
INCENP DNA
merge
Tubulin
INCENP
INCENP Tubulin
Tubulin
INCENP
INCENP
Tubulin
B
INCENP^OFF
Wild type
DNA
Tubulin
merge
3D view
DNA Tubulin
Tubulin
ST752AA
DNA
Tubulin
merge
3D view
DNA Tubulin
Tubulin

Figure S4: *Anaphase spindle midzone in INCENP wild type and ST752AA expressing cells.* **A)** Anaphase cell shown previously in Figure S3A for ST752AA mutant. We have increased the contrast and brightness slightly on the tubulin and INCENP channel so that anaphase spindle midzone is seen more clearly. A magnification of the midzone can be seen on the right-hand side of the panel. White arrow heads show clear antiparallel microtubules where INCENP is bound. Scale bars: 5 µm**.** **B)** Accurate analysis of microtubules at the spindle midzone of $INCENP^{OFF}$ cells expressing wild type or ST752AA mutant INCENP proteins during anaphase. Immunostaining of α-tubulin (green) together with DNA (DAPI, blue). Scale bars: 5 µm.

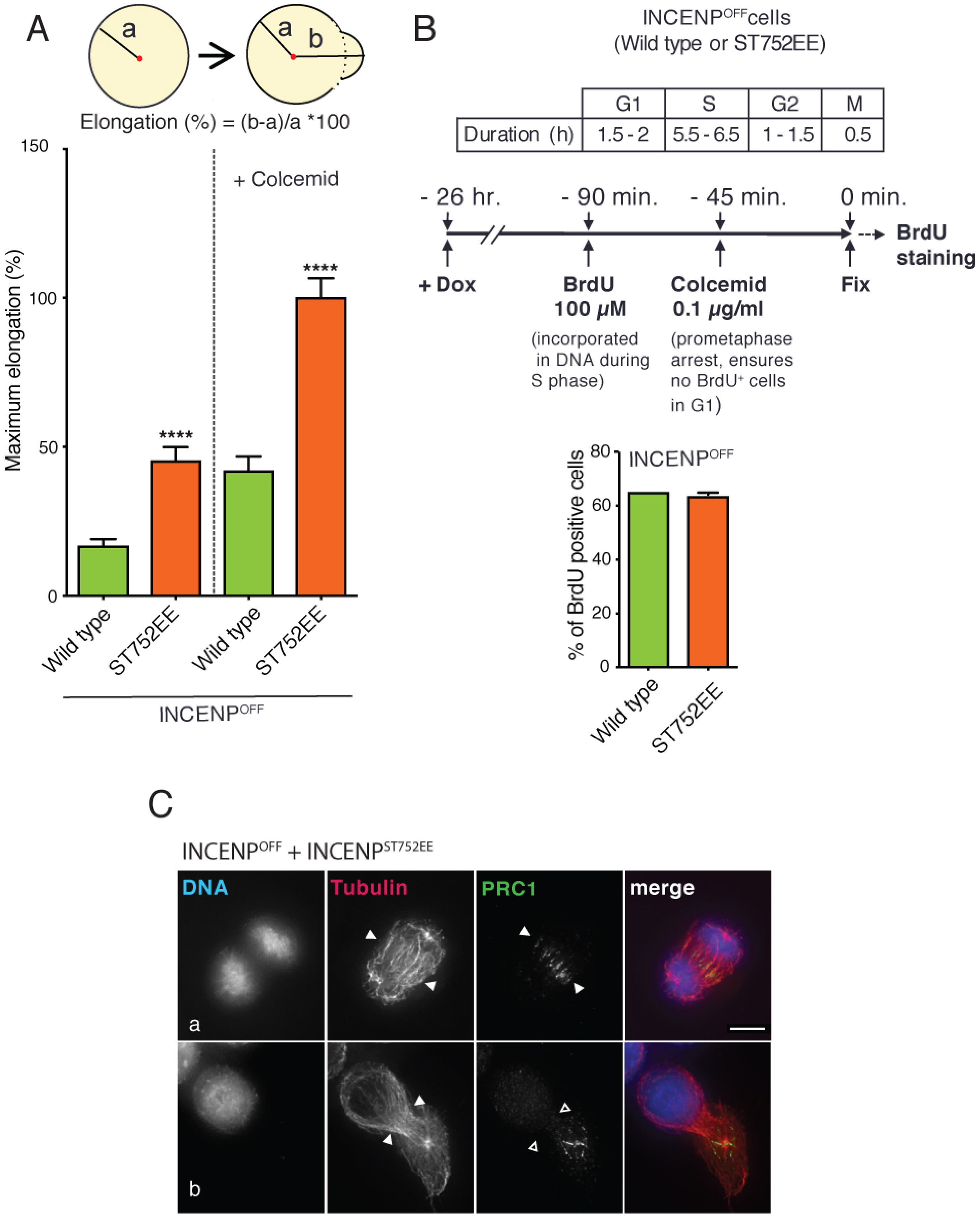

Journal of Cell Science • Supplementary information

Figure S5: *ST752EE mutant triggers the formation of ectopic cleavage furrows*. **A)** measurements of maximum elongation, as depicted on the inserted diagram, from DIC movies (frames shown in figure 7A) of INCENP^{OFF} cells expressing INCENP wild type or ST752EE mutation in the presence or absence of colcemid, a minimum of 18 cells were analyzed per condition, unpaired t-test, values are means ± SEM, two tailed P-values indicated with asterisks. **B)** schematic representation of the BrdU experiment shown in Figure 5. Quantification of BrdU positive cells in INCENP^{OFF} cells expressing either wild-type or ST752EE mutant protein, n=2 independent experiments. **C)** immunostaining of PRC1 (green) and α-tubulin (red) together with DNA (DAPI, blue) on INCENP^{OFF} cells expressing INCENP ST752EE mutant protein. White arrowheads are pointing at cleavage furrow in a and pseudo-cleavage furrow in b where PRC1 in not recruited. Scale bar: 5 µm.

Journal of Cell Science • Supplementary information

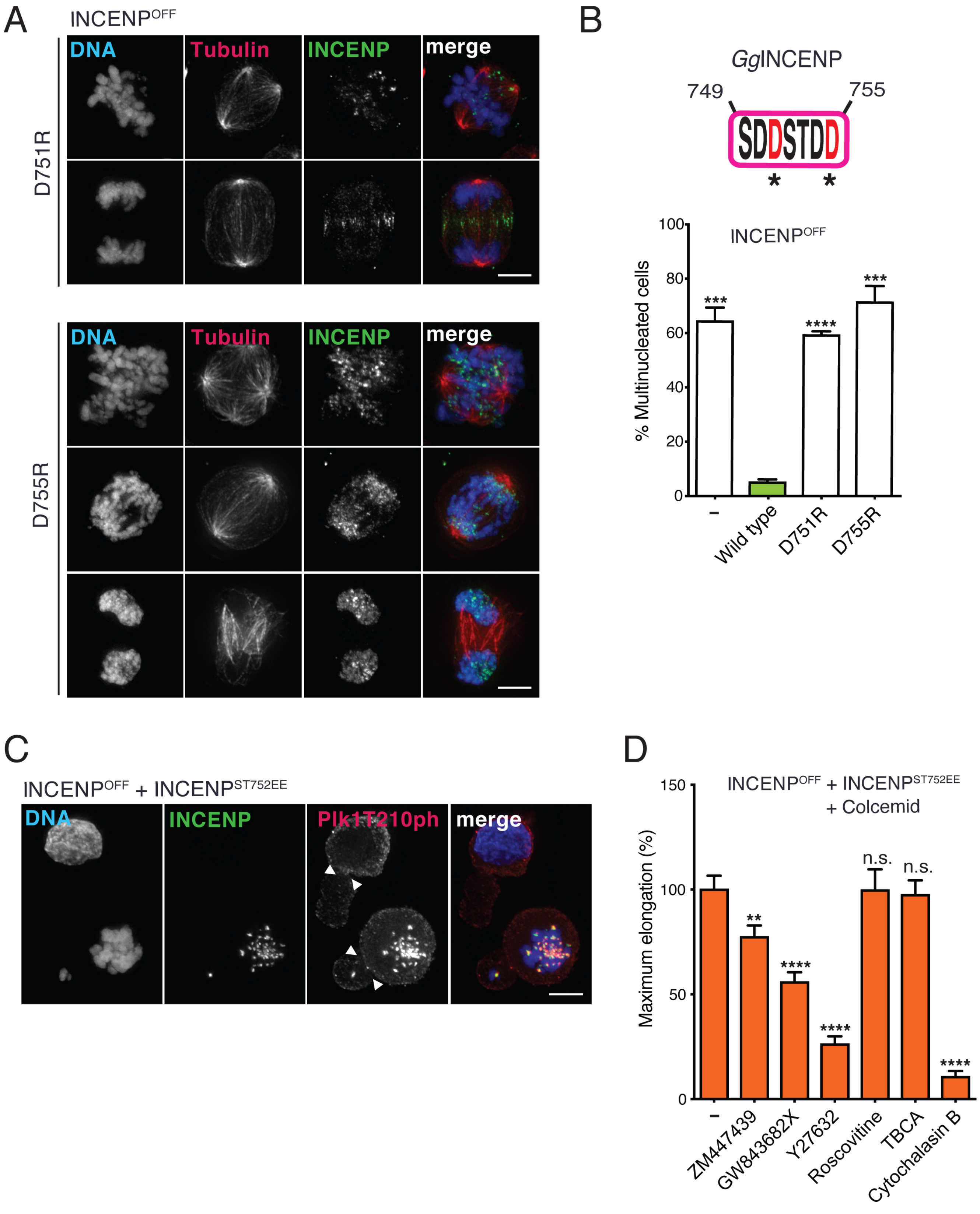

Journal of Cell Science • Supplementary information

Figure S6: *D751R and D755R INCENP mutants strongly affect cytokinesis*. *Ectopic cleavage furrows generated by ST752EE mutation are Plk1-, ROCK1- and Aurora B-dependent.* **A)** immunostaining of INCENP (green) and α-tubulin (red) together with DNA (DAPI, blue) on $INCENP^{OFF}$ cells expressing D751R and D755R mutant INCENP proteins. Scale bar: 5 µm. **B)** quantification of multinucleated $INCENP^{OFF}$ cells and $INCENP^{OFF}$ cells expressing wild type, D751R or D755R mutant INCENP proteins. Diagram showing the position of D751 and D755 within the SDDSTDD motif of *Gg*INCENP, n=3 independent experiments, unpaired t-test, values are means ± SEM, two tailed P-values are indicated with asterisks. **C)** immunostaining of INCENP (green) and Plk1T210ph (red) together with DNA (DAPI, blue) on colcemid treated $INCENP^{OFF}$ cells expressing ST752EE mutant protein. White arrowheads are pointing at ectopic membrane contractions. Scale bar: 5 µm. **D)** measurements of maximum elongation, from DIC movies of $INCENP^{OFF}$ cells expressing INCENP ST752EE treated or not with ZM 447439, GW 843682X, Y 27632, Roscovitine, TBCA or cytochalasin B, in the presence of colcemid. Unpaired t-test, values are means ± SEM, two tailed P-values indicated with asterisks, n.s.: not significant.

Journal of Cell Science • Supplementary information

SUPPLEMENTARY MOVIES

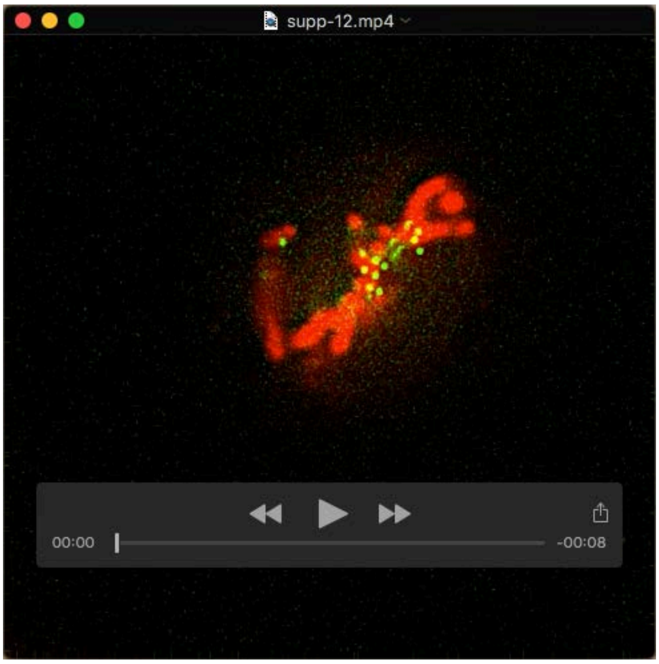

Movie 1 related to Figure 4C: INCENP^{OFF} cells expressing H2B:mRFP and CENP-H:GFP filmed from metaphase till next G1phase, related to Figure 4C. Frames are 5s apart.

Journal of Cell Science • Supplementary information

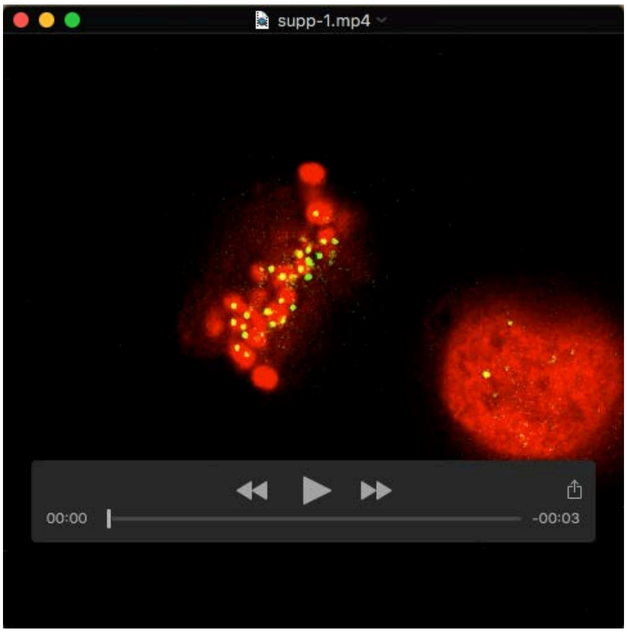

Movie 2 related to Figure 4C: INCENP^{OFF} expressing INCENP^{ST752WT} cells stably transfected with H2B:mRFP and CENP-H:GFP filmed from metaphase till next G1phase, related to Figure 4C. Frames are 5s apart.

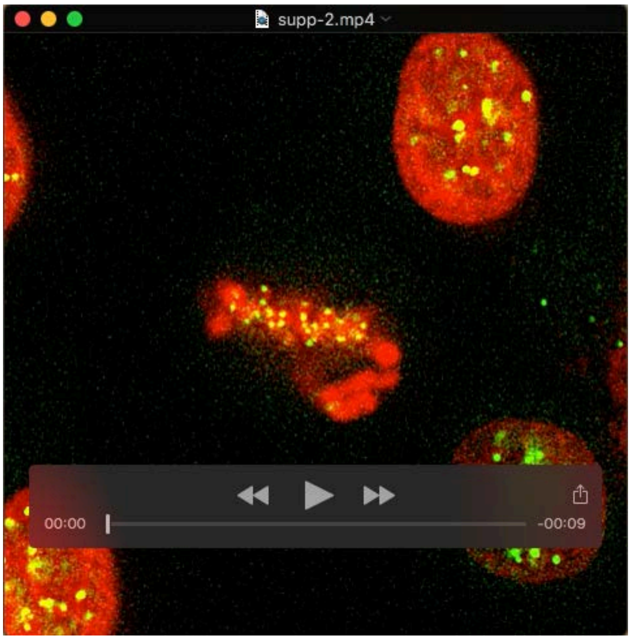

Movie 3 related to Figure 4C: INCENP^{OFF} expressing INCENP^{ST752AA} cells stably transfected with H2B:mRFP and CENP-H:GFP filmed from metaphase till next G1phase, related to Figure 4C. Frames are 5s apart.

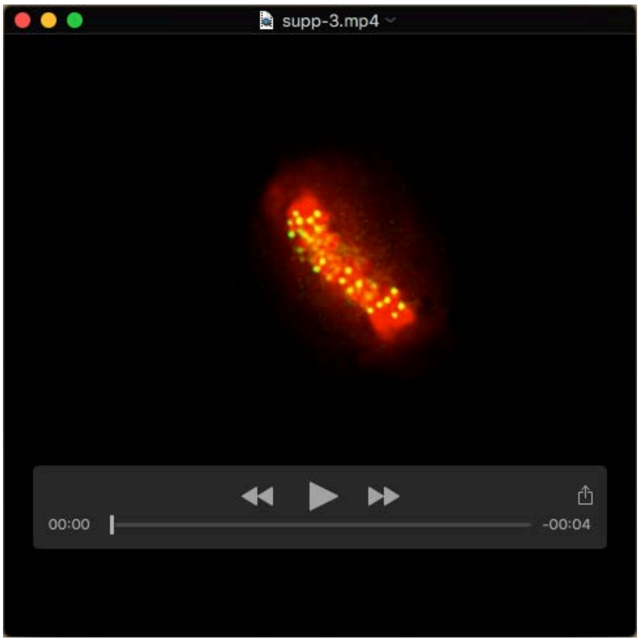

Movie 4 related to Figure 4C: INCENP^{OFF} expressing INCENP^{ST752EE} cells stably transfected with H2B:mRFP and CENP-H:GFP filmed from metaphase till next G1phase, related to Figure 4C. Frames are 5s apart.

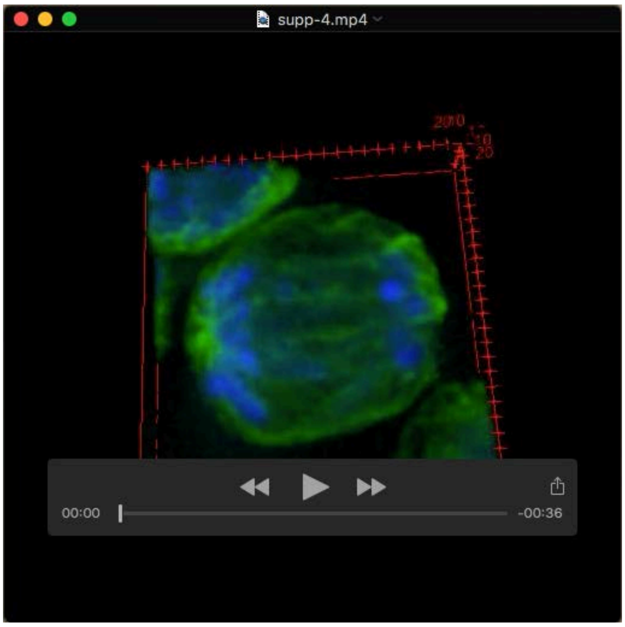

Movie 5 related to Figure S4: 3D reconstruction of INCENP^{OFF} expressing INCENP^{ST752WT} cells in anaphase. Immunostaining of α-tubulin (green) together with DNA (DAPI, blue). 3D analysis and visualization was performed using 3D viewer in Fiji and displayed as volume.

Journal of Cell Science • Supplementary information

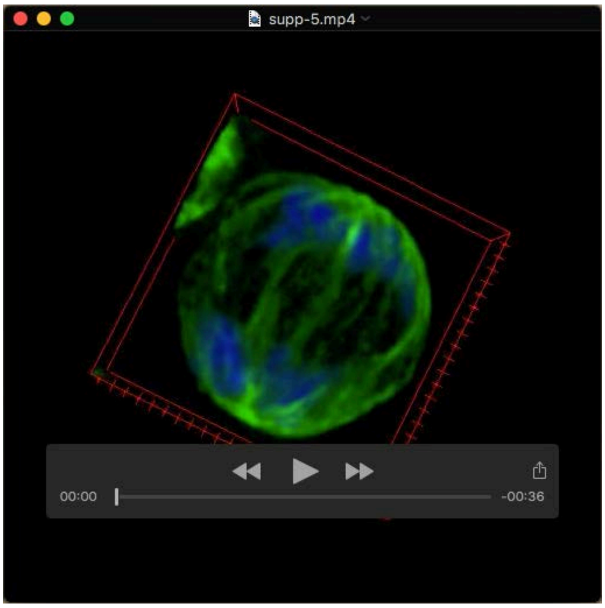

Movie 6 related to Figure S4: 3D reconstruction of INCENP^{OFF} expressing INCENP^{ST752AA} cells in anaphase. Immunostaining of α-tubulin (green) together with DNA (DAPI, blue). 3D analysis and visualization was performed using 3D viewer in Fiji and displayed as volume.

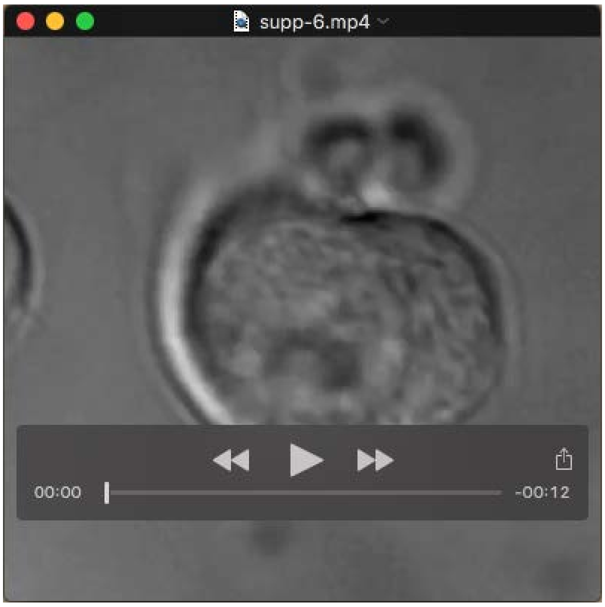

Movie 7 related to Figure 7: INCENP^{OFF} expressing INCENP^{ST752EE} cells filmed over 20 minutes in presence of colcemid, related to Figure 7. Frames are 10s apart.

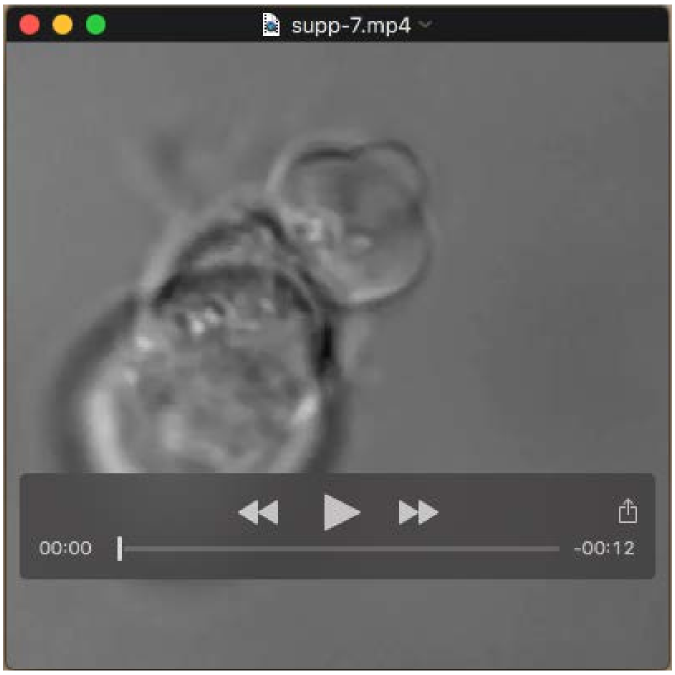

Movie 8 related to Figure 7: INCENP^{OFF} expressing INCENP^{ST752EE} cells filmed over 20 minutes in presence of colcemid and the Aurora B inhibitor ZM447439, related to Figure 7. Frames are 10s apart.

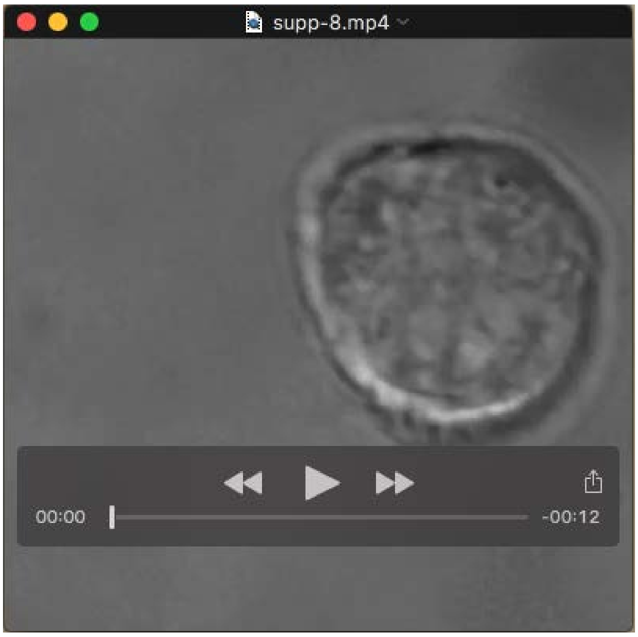

Movie 9 related to Figure 7: INCENP^{OFF} expressing INCENP^{ST752EE} cells filmed over 20 minutes in presence of colcemid and the Plk1 inhibitor GW843682X, related to Figure 7. Frames are 10s apart.

Journal of Cell Science • Supplementary information

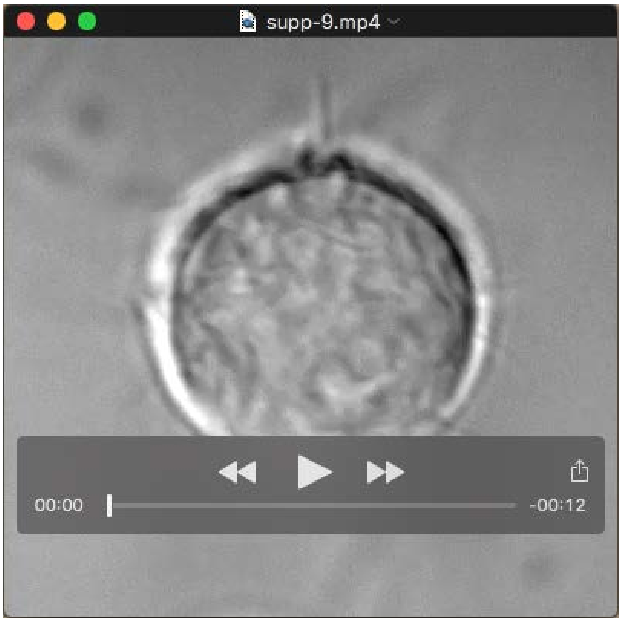

Movie 10 related to Figure 7: INCENP^{OFF} expressing INCENP^{ST752EE} cells filmed over 20 minutes in presence of colcemid and the ROCK1 inhibitor Y 27632, related to Figure 7. Frames are 10s apart.

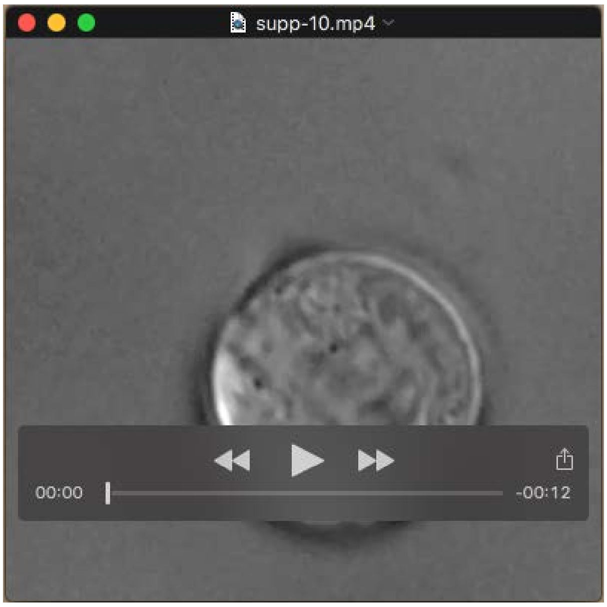

Movie 11 related to Figure 7: INCENP^{OFF} expressing INCENP^{ST752EE} cells filmed over 20 minutes in presence of colcemid and the actin inhibitor, cytochalasin B, related to Figure 7. Frames are 10s apart.
